# Differential effects of amnion and chorion membrane extracts on osteoblast-like cells due to the different growth factor composition of the extracts

**DOI:** 10.1371/journal.pone.0182716

**Published:** 2017-08-10

**Authors:** Yoon Young Go, Sung Eun Kim, Geum Joon Cho, Sung-Won Chae, Jae-Jun Song

**Affiliations:** 1 Department of Otorhinolaryngology-Head and Neck Surgery, Korea University College of Medicine, Seoul, Korea; 2 Department of Orthopedic Surgery and Rare Diseases Institute, Korea University College of Medicine, Seoul, Korea; 3 Department of Obstetrics and Gynecology, Korea University College of Medicine, Seoul, Korea; University of Massachusetts Medical School, UNITED STATES

## Abstract

Human amniotic membrane extracts contain numerous growth factors and bioactive substances. However, osteogenic effects of amnion and chorion membrane extracts (AME and CME, respectively) on osteoblasts are unclear. In this study, we explored the ability of AME and CME to promote the osteogenic differentiation of osteoblast-like MG-63 cells. MG-63 cells were cultured in osteogenic induction medium (OIM) with or without exogenous AME and CME. CME enhanced the osteogenic differentiation of MG-63 cells compared with AME, as indicated by increased mineralization; alkaline phosphatase activity; and mRNA expression of osteogenic marker genes encoding integrin-binding sialoprotein (*IBSP)*, *RUNX2*, *OSTERIX*, and osteocalcin (*OCN)*. Interestingly, AME and CME contained different combinations of osteogenesis-related growth factors, including basic fibroblast growth factor (bFGF), transforming growth factor beta-1 (TGFβ-1), and epidermal growth factor (EGF), which differentially regulated the osteogenic differentiation of MG-63 cells. bFGF and TGFβ-1 present in CME positively regulated the osteogenic differentiation of MG-63 cells, whereas EGF present in AME negatively regulated the differentiation of MG-63 cells. Moreover, exogenous treatment of EGF antagonized CME-induced mineralization of extracellular matrix on MG-63 cells. We compared the osteogenic efficacy of CME with that of BMP2, bFGF, and TGFβ-1 alone or their combinations. We observed that CME greatly enhanced osteogenesis by providing a conductive environment for the differentiation of MG-63 cells. Together, our results indicated that human AME and CME exerted differential effects on osteogenesis because of the presence of different compositions of growth factors. In addition, our results highlighted a new possible strategy of using CME as a biocompatible therapeutic material for bone regeneration.

## Introduction

Osteogenesis is a sequential process involving the proliferation and differentiation of osteogenic cells, activation of osteogenesis-specific genes, and maturation and mineralization of extracellular matrix (ECM) [1, 2]. During bone formation, osteoblasts, the major bone-forming cells, synthesize bone matrix proteins, including alkaline phosphatase (ALP), osteocalcin (OCN), and type I collagen, and subsequently produce a calcium- and phosphate-based mineral that is deposited into the ECM to form a strong, mineralized tissue [3, 4]. Osteoblast differentiation is tightly controlled by specific extracellular regulatory proteins such as growth factors, hormones, vitamins, and cytokines [5]. Growth factors and cytokines modulate cell fate, including proliferation, differentiation, survival, and migration. Particularly, growth factors play an important role in bone regeneration and remodeling and several growth factors have been used in clinical therapies for treating bone defects[6, 7], However, use of growth factors in clinical therapies is impeded by problems such as low yield of recombinant growth factors and poor efficacy and stability [8].

The human amniotic sac is derived from the placenta, which is composed of conjoined amnion (inner) and chorion (outer) membranes (AM and CM, respectively). In addition to its unique properties, the human amniotic sac contains numerous growth factors and cytokines, indicating that it is an ideal biomaterial for various therapeutic applications, including wound healing. Further, use of the human amniotic membrane in clinical therapy is associated with minimum inflammation, angiogenesis, and microbial infection [9–11]. We previously demonstrated the potential of human amniotic membrane extracts to promote the osteogenic differentiation of osteoblast-like MG-63 cells [12]. Moreover, the extracts prepared from amnion/chorion membrane have showed greater effect on osteogenesis of MG-63 cells than the extracts from a single layer of the amnion membrane. This may be because of the different growth factors present in AM and CM.

Important regulatory growth factors involved in osteogenesis include bone morphogenetic proteins (BMP2 and BMP4), basic fibroblast growth factor (bFGF), transforming growth factor beta-1 (TGFβ-1), and epidermal growth factor (EGF) [13–16]. Recently, Rui-Dong and Li showed that the combination of BMP9 and TGFβ-1 promoted the osteogenic differentiation of mesenchymal stem cells, suggesting that these growth factors exerted a synergistic effect on osteogenesis in a dose-dependent manner [17]. This finding also suggested that a crosstalk between BMP9 and TGFβ-1 signaling pathways promoted osteogenesis [17, 18]. Furthermore, several studies have indicated that many osteogenesis-related growth factors, which are inter-connected, temporally participate in the regulation of osteogenesis [19, 20].

Based on these findings, we hypothesized that AME and CME contain different compositions of growth factors, which may affect the overall osteogenic differentiation of MG-63 cells. Thus, the present study (1) investigated whether AME and CME exerted differential effects on osteogenesis, (2) analyzed the composition of osteogenesis-related growth factors in human AME and CME, and (3) determined the role of these growth factors in the osteogenic differentiation of MG-63 cells.

## Materials and methods

### Reagents and chemicals

Dulbecco's modified Eagle's medium (DMEM) was purchased from Lonza (Walkersville, MD, USA). Penicillin G–streptomycin sulfate and fetal bovine serum (FBS) were purchased from Gibco (Grand Island, NY, USA). Recombinant human bFGF, TGFβ-1, EGF, and BMP2 were purchased from Peprotech (Rocky Hill, NJ, USA), and inhibitors of FGF (SU5402), TGFβ-1 (SB505124), and EGF (PD153035) were purchased from Santa Cruz Biotechnology (Dallas, Texas, USA). These inhibitors were prepared in dimethyl sulfoxide (DMSO) and were used in growth factor inhibition assay.

### Preparation of human AME and CME

Human amniotic membrane matrixes were obtained from 3 donors who had undergone a caesarian section, with an approval from the Institutional Review Board (KUGH14239-002) of Korea University Guro Hospital (Seoul, Korea) for the purpose of this research. Donors with gestational diabetes, preeclampsia, or infectious diseases (specifically HIV 1–2, HBV, and HCV) were excluded. Written informed consent was obtained from each donor after explaining the purpose of this study.

To prepare AME and CME, AM was separated from amniotic membrane tissue. The remaining tissue was used as CM. AM and CM were washed 3 times with PBS containing antibiotics (100 U/mL penicillin and 100 U/mL streptomycin) to remove blood clots, were sliced into small pieces, frozen in liquid nitrogen, and finely ground by using a mortar and pestle. The ground membranes were mixed with PBS at 1:1 ratio of weight (g):volume (mL), were homogenized on ice for 1 h, and were centrifuged. After centrifugation, supernatants were filtered through a 0.22-μm pore size membrane. Concentrations of proteins in the supernatants were determined by performing DC protein assay (Bio-Rad, Hercules, CA, USA), and the supernatants were stored at -80°C until further use.

### Cell culture and in vitro osteogenic differentiation

MG-63 cells were cultured in DMEM supplemented with 10% FBS, 100 U/mL penicillin and 100 U/mL streptomycin. Human bone marrow derived mesenchymal stem cells (hMSCs)) were purchased from Lonza (Lonza, Walkersville, MD, USA) and maintained in DMEM supplemented with 10% FBS, 1% NEAA, 0.1% β-mercaptoethanol, 100 U/mL penicillin and 100 U/mL streptomycin, 5 mM L-glutamine (Gibco). MG-63 cells and hMSCs were maintained in a humidified atmosphere of 5% CO_2_ at 37°C.

For *in vitro* osteogenic differentiation, the cells were seeded in 24-well plates (density, 1 × 10^5^ cells/well). On reaching 90% confluency, their growth medium was replaced with osteogenic induction medium (OIM) containing DMEM supplemented with 10% FBS, 10 nM dexamethasone (Sigma, St. Louis, MO, USA), 0.2 mM ascorbic acid (Sigma), 10 mM β-glycerol phosphate (Sigma), and antibiotics (100 U/mL penicillin and 100 U/mL streptomycin) (Gibco). Thereafter, the medium was changed every 3 days.

### ALP activity

At 3 or 7 day after culture, MG-63 cells were washed with PBS and lysed in lysis buffer containing 0.5% Triton X-100. Concentrations of proteins in the cell lysates were determined using Bradford protein assay kit (Bio-Rad). Samples containing equal concentrations of proteins were used to determine ALP activity. ALP activity was measured using colorimetricSensoLyte^®^*p*NPP Alkaline Phosphatase Assay Kit (Anaspec, Fremont, CA, USA), according to the supplier’s protocol, and by reading the absorbance at 405 nm with a microplate reader.

### RNA isolation and real-time PCR

Total RNA was isolated using TRIzol reagent (Invitrogen, Carlsbad, CA, USA), according to the manufacturer's instructions. Next, 1 μg RNA was reverse transcribed in a 20-μL reaction mixture by using PrimeScript™ 1^st^ strand cDNA Synthesis Kit (Takara Bio, Tokyo, Japan), and quantitative polymerase chain reaction (RT-PCR) was performed using ABI Prism 7300 Detection System (Applied Biosystems, Foster City, CA, USA). Each 20 μL reaction mixture contained 10 μL 2× LightCycler 480 SYBR Green I Master Mix (Roche, Penzberg, Germany), 1 μL 5 pmol sense and antisense primers, 0.4 μL 50× Roxy dye (Applied Biosystems), and 1 μL cDNA. Amplification was performed using 40–45 cycles of denaturation at 95°C for 15 s and annealing at 60°C for 1 min. Relative mRNA expression was analyzed using 2^(-ΔΔCt)^ method and was normalized with that of the gene encoding glyceraldehyde-3-phosphate dehydrogenase (*GAPDH*). Specific primers used for PCR are listed in S1 Table.

### Calcium assay and Alizarin red S staining

Calcium assay and Alizarin red S staining were performed to determine ECM mineralization, as described previously [21, 22]. For the calcium assay, MG-63 cells were washed with PBS and were decalcified using 0.6 N HCl. Calcium concentration in the supernatant was measured using QuantiChrom™ Calcium Assay Kit (DICA-500; BioAssay Systems, Hayward, CA, USA), according to the manufacturer's instructions. The degree of mineralization was evaluated by measuring the absorbance at 612 nm with a microplate reader.

For Alizarin red S staining, MG-63 cells were washed with PBS and were fixed in 4% paraformaldehyde for 20 min. The cells were then washed twice with distilled water and were stained with Alizarin red S solution (Millipore, Darmstadt, Germany) for 20 min at room temperature. Finally, the cells were washed 3 times with 1 mL distilled water, and color change was evaluated using photographs. ECM mineralization was quantified by adding 1 mL 100 mM cetylpyridinium chloride (Sigma) to each well and by measuring optical density at 570 nm. Values were normalized by protein concentration.

### ELISA

Concentrations of BMP2, EGF, bFGF, and TGFβ-1 in AME and CME were quantified using respective Quantikine^®^ ELISA immunoassay kits (R&D system, Minneapolis, MN, USA). Briefly, microplate wells were pre-coated with monoclonal antibodies against human BMP2, EGF, bFGF, or TGFβ-1. Next, standards and samples were added to the microplate wells to allow growth factors to bind to the immobilized antibodies. Unbound proteins were removed by washing with wash solution, and enzyme-linked polyclonal antibodies against BMP2, EGF, bFGF, or TGFβ-1 were added to the microplate wells. Unbound antibodies were removed by washing, and a substrate solution was added to the microplate wells for color development. Concentrations of BMP2, EGF, bFGF, and TGFβ-1 in AME and CME were determined by measuring optical density at 450 nm with a microplate reader.

### Statistical analysis

Each experiment was performed using 2–3 extracts from each of the 3 donors. One-way ANOVA and Student's t-test were used to determine statistical differences among the experimental groups, with *p<0.05, **p<0.01, and ^#^*p*<0.001 being considered statistically significant. All the assays were performed at least in triplicate, and representative data were expressed as mean ± standard deviation (SD).

## Results

### Exogenous AME or CME alter the morphology of MG-63 cells during osteogenesis

**Fig 1:**
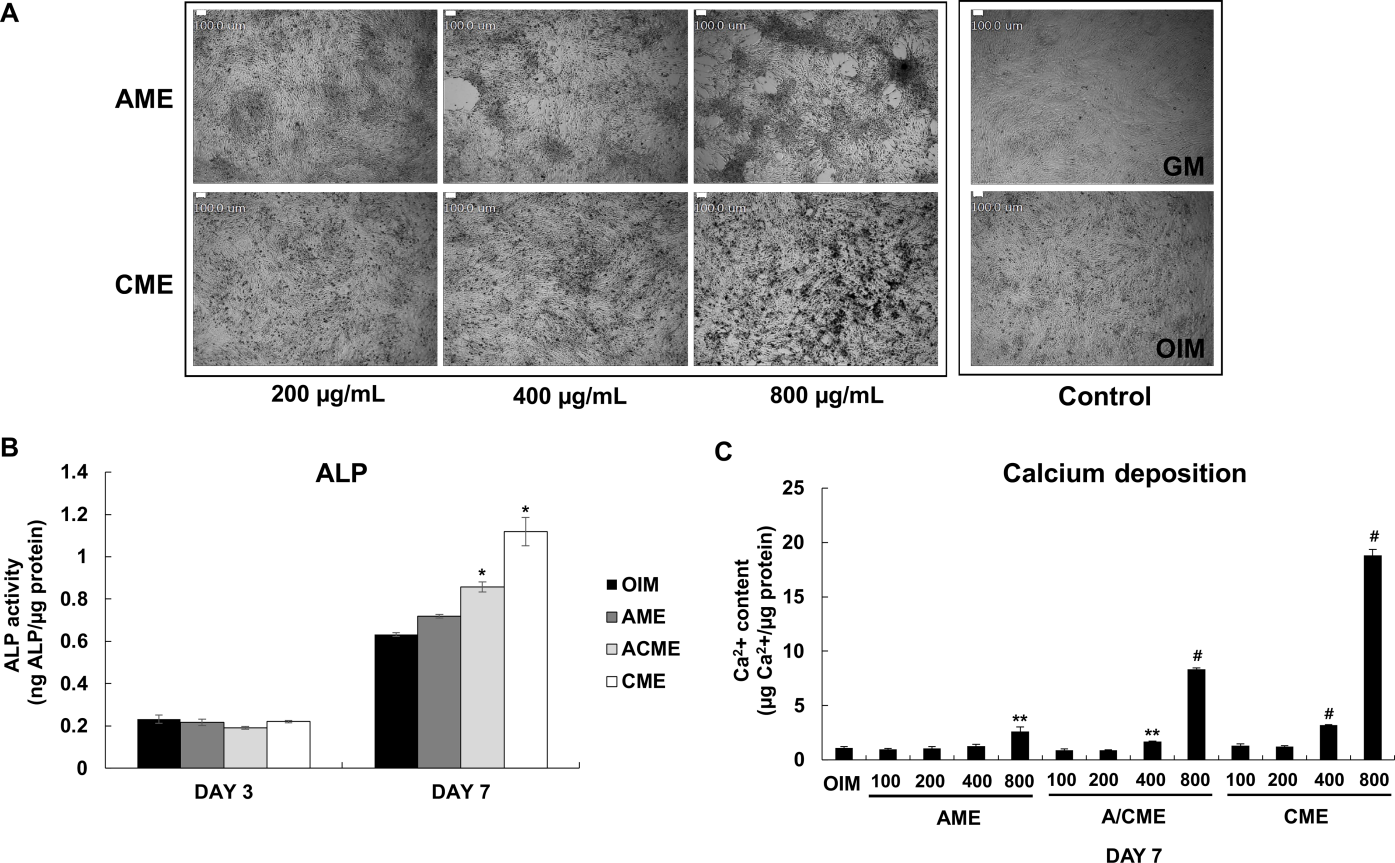
Differential effects of human amniotic membrane extract according to membrane composition during osteogenesis of MG-63 cells. (A) Confluent MG-63 cells cultured in OIM were treated with various concentrations (200–800 μg/mL) of both AME and CME. Morphological changes in the treated cells after 7 days of treatment were examined under a light microscope. Scale bars represent 100 μm. (B) MG-63 cells were cultured in OIM with or without 100 μg/mL AME, ACME, and CME. ALP activity was measured at day 3 and 7. Data are presented as the mean ± SD of multiple repeated experiments; *p< 0.05 versus OIM, indicates statistical significance. (C) MG-63 cells were cultured in OIM and AME, ACME, or CME was added in a dose-dependent manner. After 7 days, Calcium concentration was measured spectrophotometrically by using phenolsulfonephthalein dye as a substrate for free calcium. Relative calcium concentration was normalized to microgram of protein from each sample. Data are presented as the mean ± SD of multiple repeated experiments; **p< 0.01 and ^#^*p*< 0.001 versus OIM, indicates statistical significance.

Our previous studies suggested the potential ability of human amniotic membrane extracts on osteogenic differentiation of osteoblasts [12]. However, it was unclear which membrane stimulated the osteogenic differentiation of osteoblasts between AM and CM. Therefore, we separated AM and CM and prepared 2 extracts (AME and CME) in the present study (S1A and S1B Fig). To evaluate osteogenesis-related morphological changes induced by AME or CME, we cultured human osteoblast-like MG-63 cells in OIM and treated them with AME or CME in a dose-dependent manner. As expected, treatment with AME or CME induced differential morphological changes in MG-63 cells compared with those in untreated cells cultured in only growth medium (GM) or only OIM for 7 days (Fig 1A, right square images). While cells cultured in only OIM showed initiation of osteogenic differentiation compared with cells cultured in only GM, cells cultured in OIM containing CME showed more obvious evidence of osteogenesis than only OIM condition (Fig 1A, left square image). However, no detectable evidence of mineralized nodule formation was observed in cells cultured in OIM containing AME; however, these cells showed excessive cell migration and accumulation (Fig 1A, left square image).

### Potential of CME for promoting osteogenic differentiation

**Fig 2:**
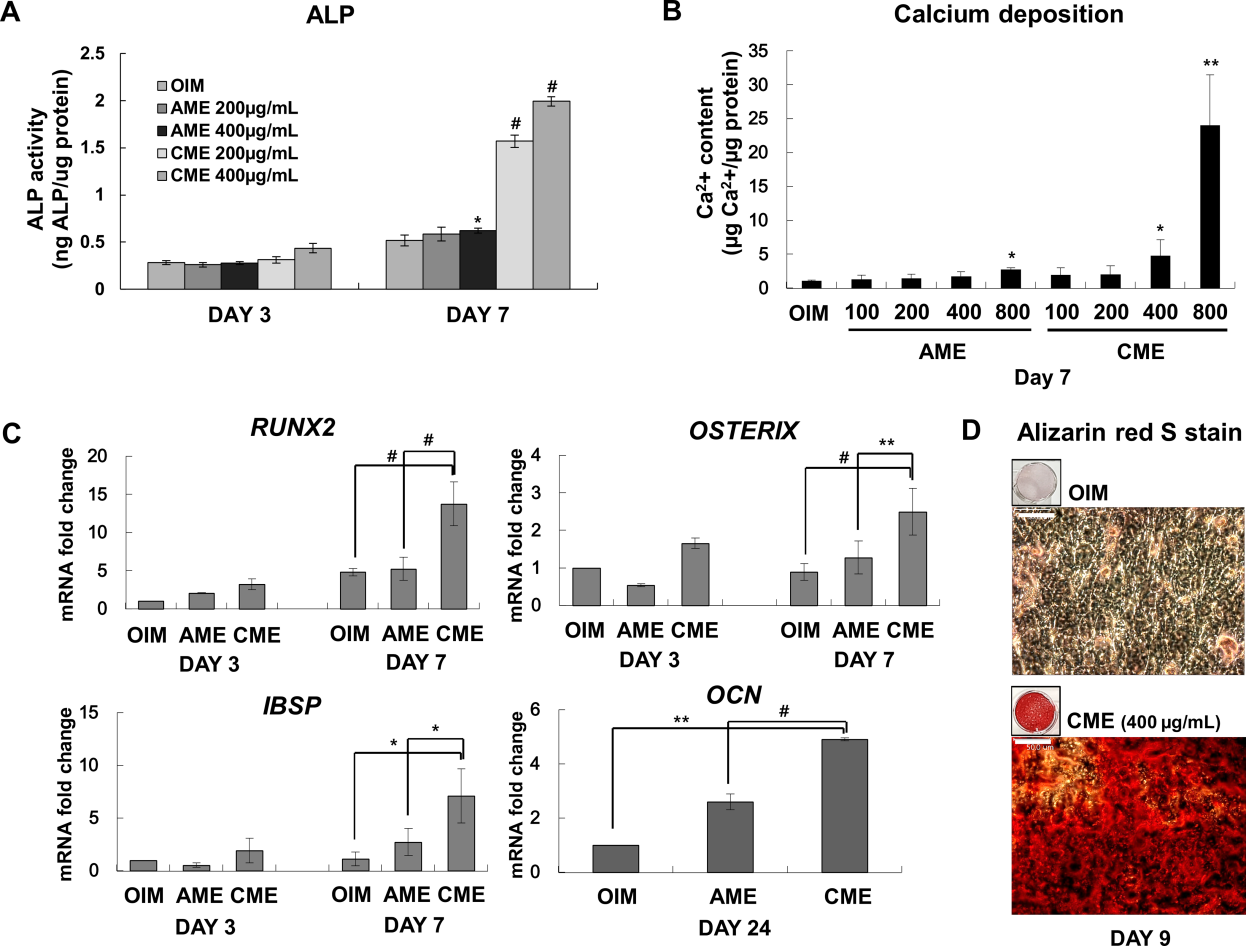
Potent ability of CME to promote the osteogenic differentiation of MG-63 cells. (A) ALP activity of MG-63 cells cultured in OIM with or without AME or CME (200–400 μg/mL) for 3 or 7 days was measured. *p< 0.05 and ^#^*p*< 0.001 versus OIM of day 7. (B) MG-63 cells were cultured in OIM, and AME or CME was added in a dose-dependent manner. Calcium concentration after 7 days was measured. *p< 0.05 and **p< 0.01 versus OIM. (C) MG-63 cells were cultured in OIM with or without 200 μg/mL of AME or CME to evaluate the expression of osteogenic marker genes by quantitative real-time RT-PCR. The mRNA levels of 3 genes encoding *RUNX2*, *OSTERIX*, and *IBSP* were determined on days 3 and 7. Expression of the gene encoding *OCN*, which is expressed by osteoblasts in the late stage of osteogenic differentiation, was analyzed on day 24. Results of gene expression were normalized with those obtained for *GAPDH* and were presented as fold change. *p< 0.05, **p< 0.01, and ^#^*p*< 0.001, Student’s t-test. (D) MG-63 cells grown in OIM and treated with 400 μg/mL CME were cultured for 9 days and were stained with Alizarin red S. Control cells were cultured in only OIM. Scale bars represent 500 μm. Data are presented as the mean ± SD of multiple repeated experiments.

Next, we examined whether AME and CME differentially promoted the osteogenic differentiation of MG-63 cells. To measure ALP activity, MG-63 cells were cultured in OIM with or without AME, ACME (amnion/chorion membrane extracts) or CME for 3 or 7 days, respectively. On day 7, ALP activity was slightly different in cells treated with AME, ACME and CME at the concentration of 100 μg/mL compared with that in cells cultured in only OIM (Fig 1B) while a much higher concentration (200 and 400 μg/mL) was clearly determine the significantly higher effect of CME on ALP activity than AME (Fig 2A). In addition, CME has potential ability for promoting osteogenic differentiation when we compared the result of AME and ACME. Calcium concentration also significantly increased by approximately 25 folds in cells treated with 800 μg/mL CME (Fig 1C and Fig 2B). In contrast, calcium concentration in cells treated with the same concentration of AME was only 2- to 3-fold higher than that in cells cultured in only OIM (Fig 1C and Fig 2B). CME treatment also significantly upregulated the expression of osteogenic marker genes encoding *RUNX2* (8.939 fold), *OSTERIX* (1.607 fold), and *IBSP* (6.101 fold, *p =* 0.0292) on day 7 and the gene encoding *OCN* (3.907 fold, *p =* 0.0053) on day 24 (Fig 2C). Although AME treatment upregulated the expression of these osteogenic marker genes, the upregulation was not significant. Alizarin red S staining showed a strong increase in the mineralization of MG-63 cells treated with 400 μg/mL CME compared with that of cells cultured in only OIM (Fig 2D). These results suggested that CME but not AME markedly stimulated the ALP activity, expression of osteogenic marker genes, and mineralization of MG-63 cells during osteogenesis.

### Effect of suramin sodium on the inhibition of the osteogenic differentiation of CME-treated MG-63 cells

**Fig 3:**
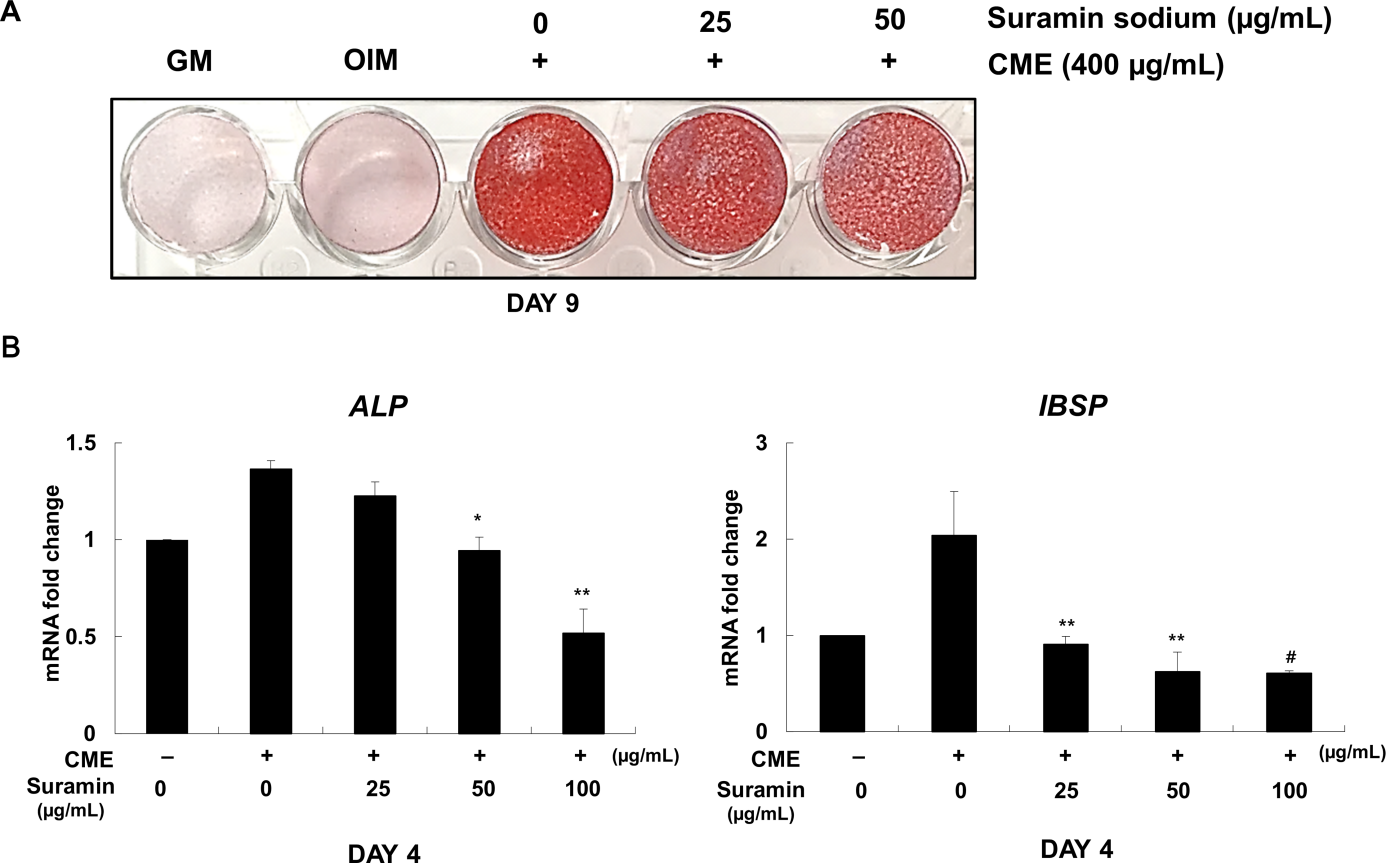
Suramin sodium inhibited the effectiveness of CME in promoting the osteogenic differentiation of MG-63 cells. (A) MG-63 cells were seeded in GM. On the next day, GM was replaced with OIM containing various concentrations of suramin sodium (0–50 μg/mL). After 1 h, the cells were treated with 400 μg/mL CME. After 9 days, mineralization of MG-63 cells was determined by performing Alizarin red S staining. (B) MG-63 cells were pretreated with suramin sodium (0–100 μg/mL) for 1 h, followed by treatment with 200 μg/mL CME. Expression of genes encoding *ALP* and *IBSP* was determined by quantitative real-time RT-PCR on day 4. *p< 0.05, **p< 0.01, and ^#^*p*< 0.001 versus only CME. Data are presented as the mean ± SD of multiple repeated experiments.

To determine whether growth factors present in CME enhanced the osteogenesis of MG-63 cells, we examined the inhibitory effect of suramin sodium, which is known to block the interaction of various growth factors such as bFGF, PDGF, EGF, and TGFβ-1 with their respective surface receptors [23–25],on MG-63 cells cultured in OIM supplemented with CME. Expectedly, dose-dependent pretreatment of suramin sodium in CME-treated MG-63 cells gradually impaired their mineralization, as indicated by Alizarin red S staining (Fig 3A) and decrease in the mRNA levels of genes encoding *ALP* and *IBSP* during the early stage of osteogenic differentiation (Fig 3B). Suramin sodium did not affect the osteogenic differentiation of MG-63 cells cultured in only OIM (S3A Fig). These results suggested that growth factors present in CME were involved in promoting the osteogenesis of MG-63 cells.

### Concentrations of osteogenesis-related growth factors in AME and CME

**Fig 4:**
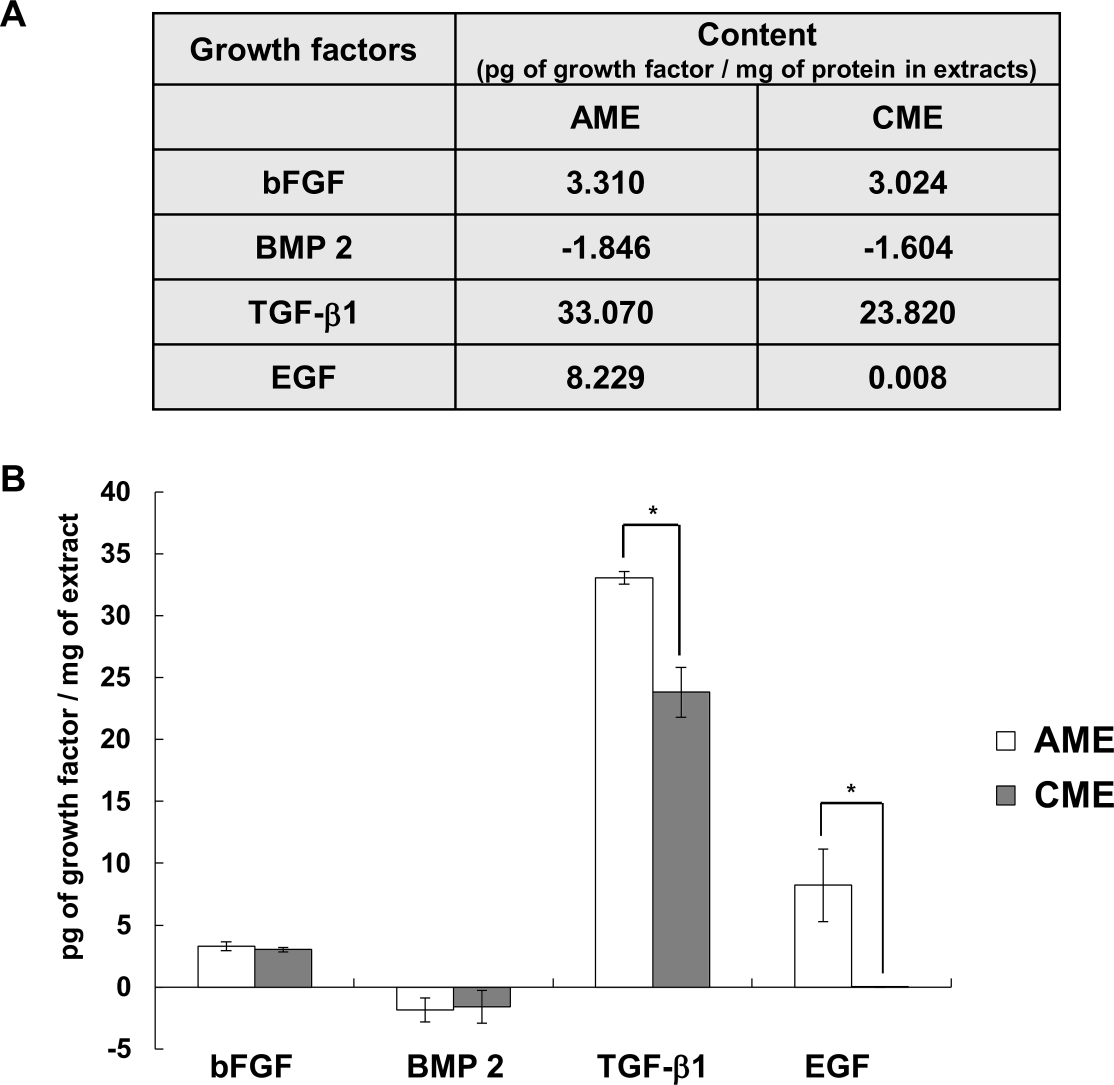
Concentrations of different osteogenesis-related growth factors in AME and CME. (A) A table showing the relative concentrations of growth factors in AME and CME (picogram of growth factor/milligram of AME or CME). (B) Histograms showing the distribution of growth factor concentrations in the two membrane extracts from the 3 donors. Data are presented as the mean ± SD of 2 repeated experiments; *p< 0.05, Student’s t-test, indicates statistical significance.

It has been known that bFGF, EGF and TGFβ-1 are involved in the proliferation and differentiation of osteogenic-lineage cells. However, the stage of osteogenesis in which these growth factors act on is still unclear [6, 14, 16, 17, 26, 27]. We next evaluated the concentrations of osteogenesis-related growth factors, including bFGF, TGFβ-1, BMP2, and EGF, in AME and CME by performing ELISA. Results presented in Fig 3 indicated that the concentrations of bFGF and TGFβ-1 were comparable between AME and CME even though AME contained higher concentrations of TGFβ-1 than CME. Interestingly, BMP2, which is currently used to stimulate bone regeneration [28], was not present in both the extracts while EGF was present only in AME (Fig 4).

### Inhibitory effect of SU5402 and SB505124 on the osteogenic differentiation of CME-treated MG-63 cells

**Fig 5:**
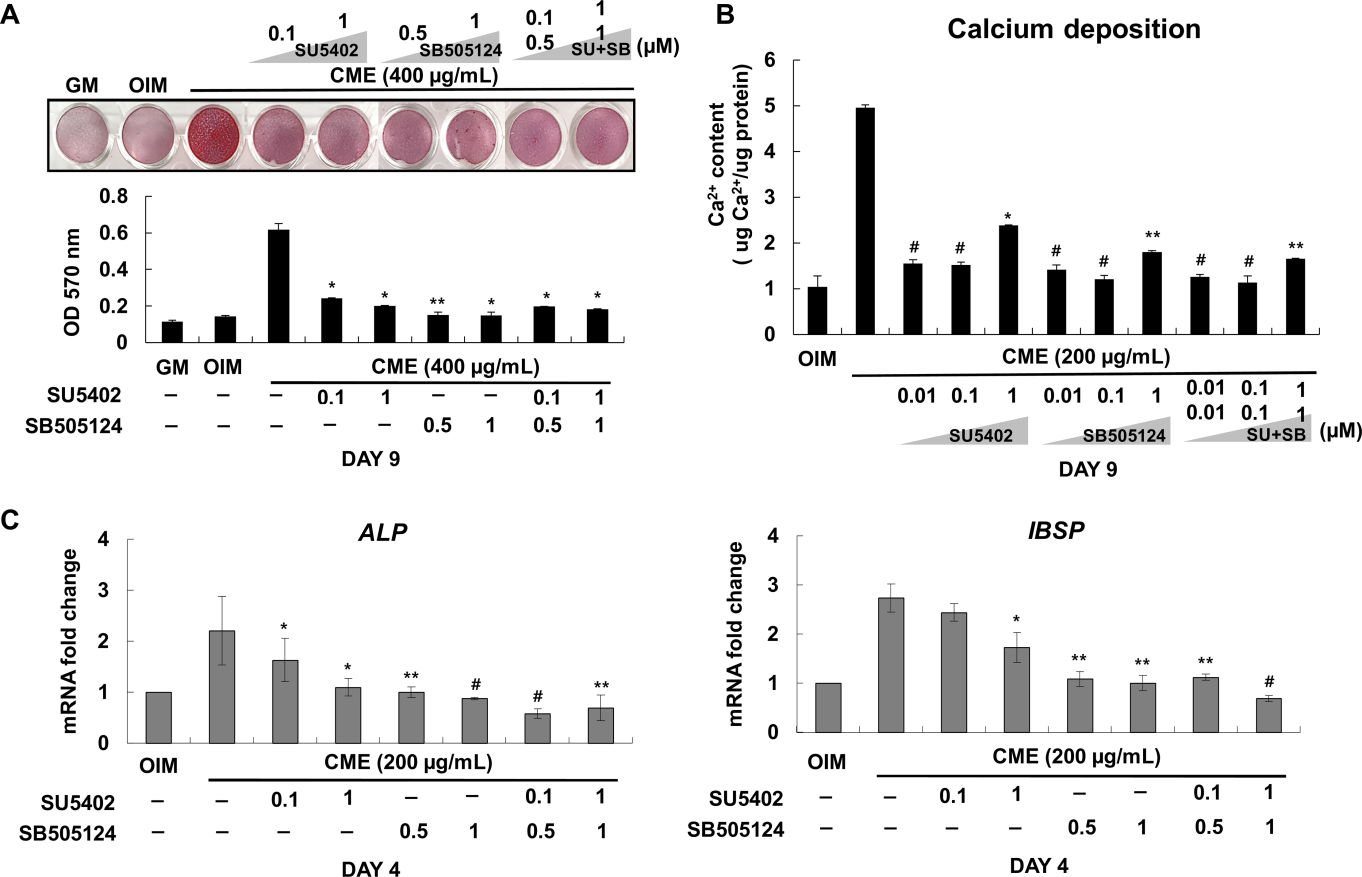
SU5402 and SB505124 inhibited the effect of CME on the osteogenic differentiation of MG-63 cells. (A) MG-63 cells were cultured in OIM supplemented with DMSO (0.1% v/v) and were pretreated with various concentrations of SU5402 (0.1–1 μM) or SB505124 (0.5–1 μM) for 1 h, followed by treatment with 400 μg/mL CME. After 9 days, mineralization was determined by performing Alizarin red S staining. Alizarin red S stain was extracted using 10% cetylpyridinium chloride, and absorbance was measured at 570 nm. (B) For the calcium assay, the cells were pretreated with DMSO (0.1% v/v) and various concentrations of SU5402 (0.1–1 μM) or SB505124 (0.5–1 μM) alone or their combination for 1 h, followed by treatment with 200 μμg/mL CME. Calcium assay was performed after 9 days. (C) Expression of genes encoding *ALP* and *IBSP* was determined on day 4 by performing quantitative real-time RT-PCR under the experimental condition described in (A): SU5402: FGF specific inhibitor, SB505124: TGFβ-1 specific inhibitor, SU+SB: SU5402+SB505124. Data are presented as the mean ± SD of multiple repeated experiments; *p< 0.05, **p< 0.01, and ^#^*p*< 0.001 versus only CME, indicate statistical significance.

To investigate whether bFGF and TGFβ-1 present in CME positively regulated osteogenesis, we used SU5402 and SB505124, respectively, to inhibit these growth factors. Results presented in Fig 4 indicate that CME-induced ECM mineralization was completely abolished after treatment with SU5402 and SB505124 alone or their combination (Fig 5A). Calcium assay also showed that continuous treatment of CME-treated MG-63 cells with bFGF and TGFβ-1 inhibitors suppressed calcium deposition during osteogenic differentiation (Fig 5B), which was consistent with the results of Alizarin red S staining. Calcium deposition was more effectively inhibited by SB505124 than by SU5402; moreover, stable suppression of calcium deposition was observed in cells treated with a combination of SU5402 and SB505124 (Fig 5B). SU5402 and SB505124 did not affect the osteogenic differentiation of MG-63 cells at the same experimental condition (S3B and S3C Fig). We considered that MG-63 cells cultured for 4 days were in between the early and middle stages of osteogenic differentiation because these cells rapidly reached the late stage of osteogenic differentiation about 10 days of culturing in OIM supplemented with 200 μg/mL CME. Therefore, quantitative real-time RT-PCR analysis performed to evaluate the expression of early osteogenic marker genes such as those encoding *ALP* and *IBSP* on day 4. The mRNA levels of these genes was elevated in cells treated with CME but was dramatically decreased in cells treated with SU5402 and SB505124 (Fig 5C). These results clearly indicated that bFGF and TGFβ-1 present in CME positively regulated the expression of osteogenic marker genes and mineralization of the ECM during osteogenesis.

### Stimulatory effect of PD153035 on the osteogenic differentiation of AME-treated MG-63 cells

**Fig 6:**
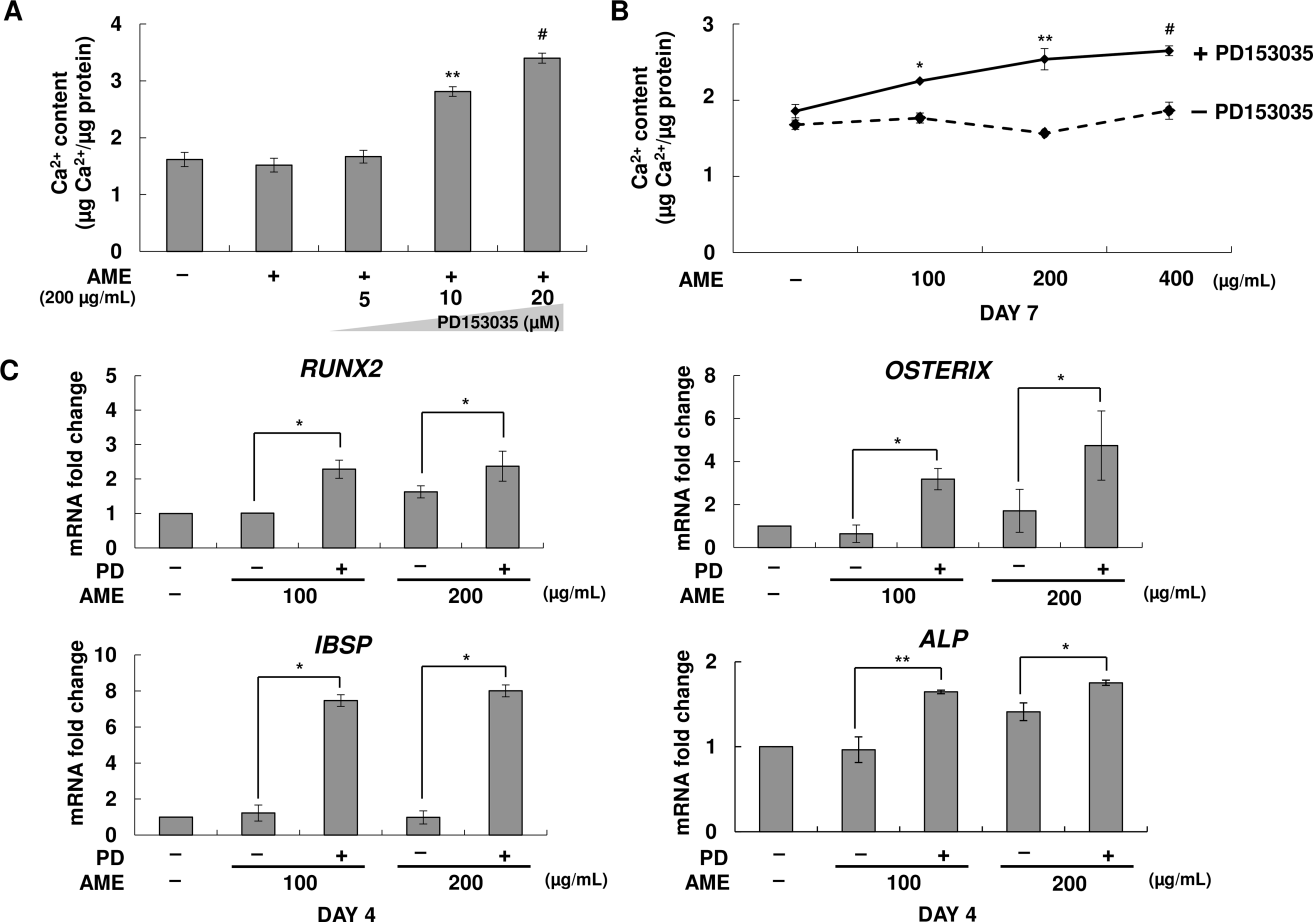
Stimulatory effect of PD153035 on the osteogenic differentiation of AME-treated MG-63 cells. (A) MG-63 cells were pretreated with DMSO (0.1% v/v) and PD153035 (5–20 μM) for 1 h. Next, the cells were treated with 200 μg/mL AME for 7 days. Calcium concentration was determined by performing the calcium assay. Data are represented as mean ± SD. **p< 0.01 and ^#^*p*< 0.001 versus only AME. (B) MG-63cells were pretreated with DMSO (0.1% v/v) and PD153035 (10 μM) for 1 h. The cells were then treated with different concentrations (100–400 μg/mL) of AME and were cultured for 7 days. Calcium concentration was determined by performing the calcium assay. Data are represented as mean ± SD. *p< 0.05, **p< 0.01, and ^#^*p*< 0.001 versus only AME. (C) Expression of genes encoding *RUNX2*, *OSTERIX*, *ALP*, and *IBSP* in cells pretreated with DMSO (0.1% v/v) and PD153035 (10 μM) for 1 h and treated with 100–200 μg/mL AME was determined after 4 days by performing quantitative real-time RT-PCR: PD: PD153035, EGF specific inhibitor. *p< 0.05 and **p< 0.01, Student’s t-test. Data are presented as the mean ± SD of multiple repeated experiments.

Next, we determined the effect of EGF present in AME on the osteogenesis of MG-63 cells. MG-63 cells cultured in OIM were pretreated with EGF-specific inhibitor PD153035, followed by treatment with the 200 μg/mL of AME. We observed that inhibition of EGF present in AME markedly increased calcium concentration (Fig 6A). Treatment with a high concentration of AME (400 μg/mL) did not affect calcium deposition on day 7 compared with that in untreated cells. In contrast, treatment with PD153035 dramatically increased the mineralization of MG-63 cells (Fig 6B) under the same experimental condition. Moreover, mRNA levels of osteogenic marker genes were significantly increased in PD153035-pretreated MG-63 cells (Fig 6C). PD153035 did not affect the osteogenesis of MG-63 cells, which were confirmed by calcium assay (S3D Fig). These results suggested that EGF present in AME inhibited the osteogenesis of MG-63 cells.

### EGF strongly suppressed the osteogenic effects of CME

**Fig 7:**
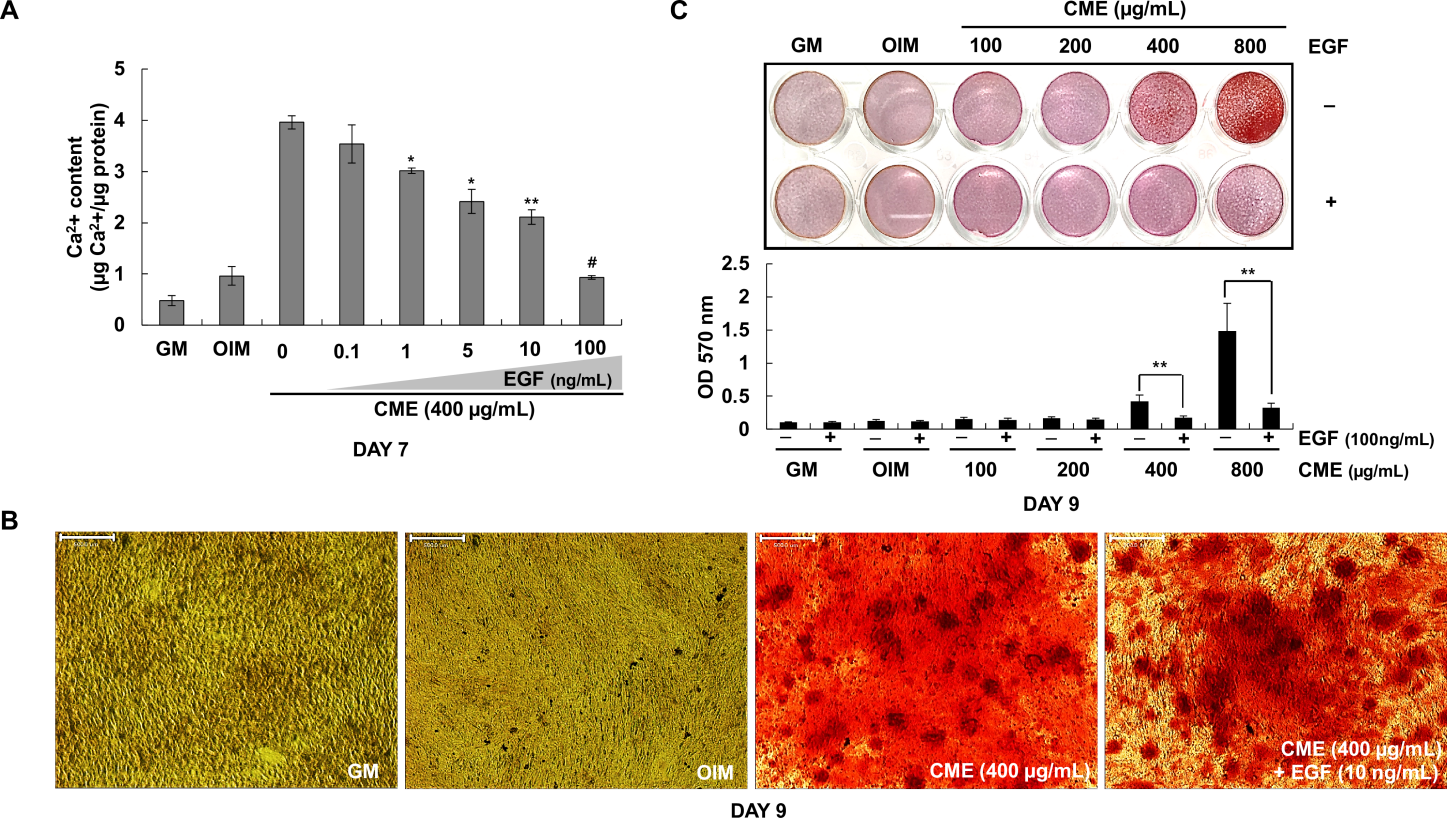
EGF strongly suppressed the osteogenic effects of CME. (A) MG-63 cells were cultured in OIM with CME (400 μg/mL) and various concentrations of EGF (0.1–100 ng/mL). Calcium assay was performed after 7 days. *p< 0.05, **p< 0.01, and ^#^*p*< 0.001 versus only CME. (B) MG-63 cells were cultured in OIM containing different concentrations of CME (100–800 μμg/mL) in the presence or absence of EGF (100 ng/mL). Mineralization rate was determined by performing Alizarin red S staining. **p< 0.01, Student’s t-test. (C) MG-63 cells were cultured in OIM containing 400 μg/mL CME with or without EGF (10 ng/mL). After 9 days, the cells were stained with Alizarin red S to determine mineralization. Scale bars represent 500 μm. Data are represented as mean ± SD of multiple repeated experiments.

Results presented in Fig 4 indicated that AME contained various growth factors such as EGF, bFGF, and TGFβ-1. However, AME treatment showed less stimulation of mineralization on MG-63 cells compared with CME treatment. CME contained appropriate concentrations of bFGF and TGFβ-1 but lacked EGF. Next, the effect of EGF concentration (0.1–100 ng/mL) on osteogenic differentiation was examined in the presence of 400 μg/mL CME. Fig 7A shows that the stimulatory effect of CME was significantly inhibited by EGF at concentrations as low as 1 ng/mL. Fig 7B similarly shows that 10 ng/mL EGF inhibited the formation of mineralized nodules in MG-63 cell culture. We observed that 10 ng/mL EGF completely inhibited CME-induced mineralization by MG-63 cells at CME concentrations ranging from 400–800 μg/mL (Fig 7C). Taken together, these data suggested that exogenous EGF treatment strongly suppressed calcium formation and ECM mineralization during the osteogenesis of CME-treated MG-63 cells.

### Strong effects of CME on human MSCs during osteogenesis

**Fig 8:**
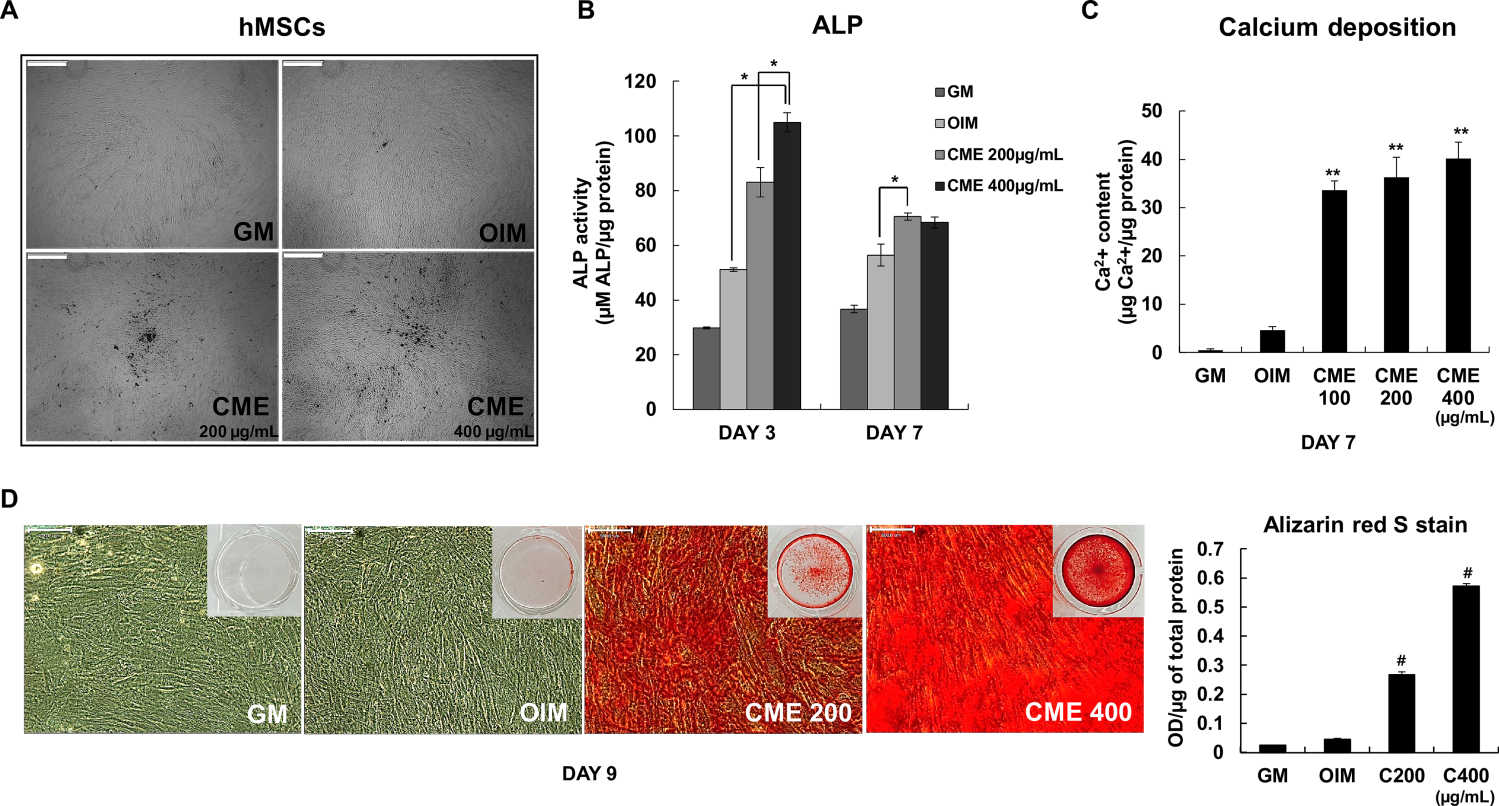
Strong effects of CME on human MSCs during osteogenesis. (A) Morphological changes in response to treatment with CME (200 and 400 μg/mL) were examined at day 7 under a light microscope. Scale bars represent 500 μμm. (B) ALP activity of hMSCs cultured in OIM with or without CME was measured at day 7. *p< 0.01, Student’s t-test. (C) Confluent hMSCs cultured in OIM were treated with various concentration of CME (100–400 μμg/mL). Calcium concentration was determined by performing the calcium assay at day 7. **p< 0.01 versus only OIM. (D) Mineralization rate was determined by performing Alizarin red S staining. Scale bars represent 500 μm. Data are presented as the mean ± SD of multiple repeated experiments; *p< 0.01 and **p< 0.001 versus OIM, indicates statistical significance.

Next, we asked if CME could stimulate human mesenchymal stem cells (hMSCs) which are used as standard cell types for therapy. The morphological change, ALP activity, calcium contents and mineralization of hMSCs were significantly promoted under the OIM condition with CME (Fig 8). But most important of all, ALP activity of hMSCs has already stimulated in response to CME treatment with a peak level detected at day 3 (Fig 8B) compared with ALP activity of MG-63 cells (Fig 2A), suggesting the strong efficacy of CME on the osteogenesis of hMSCs. Taken together, these results provide clear evidence that CME could be a biocompatible therapeutic material for bone regeneration as a strong stimulator of osteogenesis on hMSCs.

### Comparison of osteogenic effect of growth factors with that of CME

**Fig 9:**
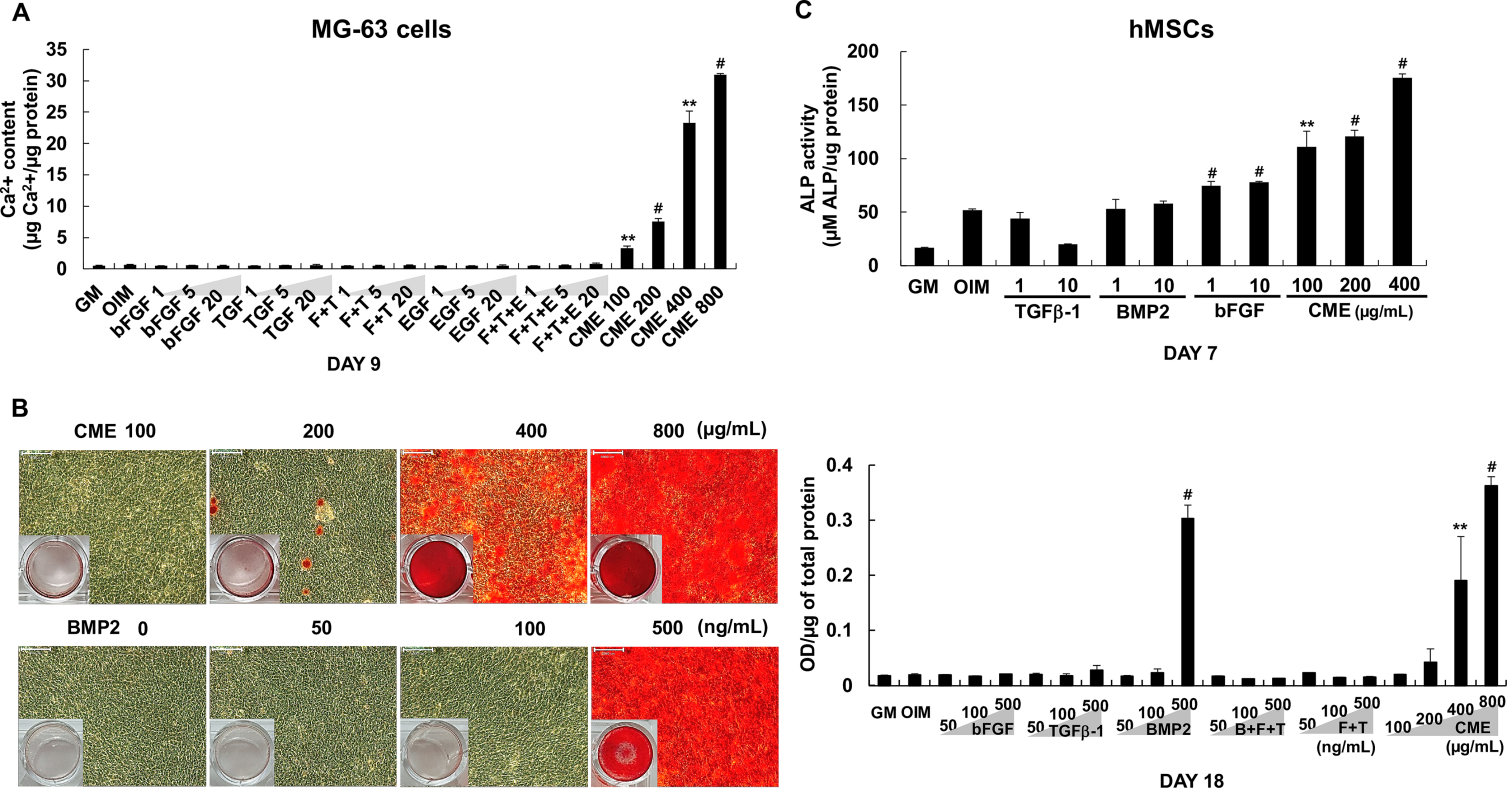
Comparison of the osteogenic effect of different growth factors with that of CME. (A) MG-63 cells grown in OIM with or without various concentrations (1, 5 and 20 ng/mL) of bFGF, TGF β-1, EGF alone or their combination and CME (100–800 μg/mL). On day 9, Calcium concentration was compared by performing the calcium assay: F+T: bFGF + TGFβ-1, F+T+E: bFGF + TGFβ-1+ EGF. (B) MG-63 cells were cultured in OIM with or without high concentration of BMP2 (50–500 ng/mL), bFGF (50–500 ng/mL), and TGFβ-1 (50–500 ng/mL) alone or their combinations (bFGF + TGFβ-1 and bFGF + TGFβ-1+ BMP2) and CME (100–800 μg/mL). The cells were treated with the indicated concentrations of the growth factors and CME every 2–3 days and were cultured for 18 days. ECM mineralization was determined by performing Alizarin red S staining and by measuring the absorbance of solubilized Alizarin red S at 570 nm: F+T: bFGF + TGFβ-1, F+T+B: bFGF + TGFβ-1+ BMP2. Scale bars represent 100 μm. (C) Human MSCs were cultured in OIM with or without various concentrations (1 and 10 ng/mL) of bFGF, TGFβ-1, BMP2 and CME (100–400 μg/mL). ALP activity was measured at day 7. Data are presented as the mean ± SD of multiple repeated experiments; **p< 0.01 and ^#^*p*< 0.001 versus OIM, indicates statistical significance.

To compare the osteogenic effect of several bone-specific growth factors and CME, we selected growth factors, bFGF, TGFβ-1, EGF and BMP2 which are involved in differentiation of osteoblasts [29] and then tested on MG-63 cells and hMSCs with CME. In Fig 9A, bone-related growth factors alone or their combination did not affect calcium formation of MG-63 cells at day 9. The ALP activity of hMSCs was slightly increased in treated with bFGF and decreased in treated with TGFβ-1 at day 7 after the induction of osteogenesis (Fig 9B). However, CME remarkably stimulated the differentiation and mineralization compared with both MG-63 cells and hMSCs (Fig 9A and 9B). Especially, BMP2 are known to highly induce the osteogenic differentiation of osteoblasts [30–33] but low concentration or the short period treatment did not exert osteogenesis in our study. To further confirm the comparison of osteogenic effect between growth factors and CME, we treated high concentration of recombinant BMP2, bFGF, and TGFβ-1 alone or their combinations and CME underlying the osteogenic condition. Analysis after 18 days of treatment with the growth factors or CME showed that treatment with high concentration of BMP2 (500 ng/mL) increased the mineralization of MG-63 cells, as confirmed by Alizarin red S staining (Fig 9C). In contrast, no mineralization was detected in cells treated with high concentrations (500 ng/mL) of bFGF and TGFββ-1, indicating that these growth factors did not exert osteogenic effect independently (Fig 9C and S4 Fig). Surprisingly, treatment with low concentration (100 μg/mL) of CME strongly induced the osteogenic differentiation of MG-63 cells under the same experimental condition. Treatment with exogenous bFGF + TGFβ-1 and bFGF + TGFβ-1+ BMP2 did not exert any synergistic effect on the osteogenic differentiation of MG-63 cells (Fig 9C and S4 Fig). Interestingly, the osteogenic effect of BMP2 was abolished when it was administered in combination with bFGF + TGFβ-1 (Fig 9C and S4 Fig), implying that this was not an optimal combination for promoting the osteogenesis of MG-63 cells.

## Discussion

The human amniotic membrane has been used as a natural scaffold in tissue engineering since the early 20^th^ century [34]. AM is a strong, flexible, and translucent biomaterial scaffold containing various growth factors and cytokines that confer it with unique anti-scarring and anti-inflammatory properties [35]. The natural AM is modified by decellularization to obtain an acellular AM matrix for clinical use. Decellularization involves the removal of epithelial cells from the surface of AM by physiological or chemical methods to eliminate immunogenicity [36, 37]. Koizumi et al. reported that the most growth factors present on the epithelium of AM are lost during decellularization [38]. Although human CM contains more growth factors and cytokines than those present on an equal surface area of AM, CM has not been used as a scaffold because it is structurally difficult to operate during decellularization compared with AM. In this study we prepared human amniotic membrane matrix-extract and evaluated the osteogenic differentiation of osteoblast-like cells. The AM matrix-extract is easy to use and storage in clinical setting and the procedure mentioned in this study did not affect the growth factors present in intact amnion and chorion membranes. Moreover, we also evaluated the less studies human CM extract’s effect on the osteogenic differentiation of osteoblasts.

Bone regeneration is a multistep process characterized by cell migration, coupling, proliferation and differentiation and involves several cells types such as osteoblasts, osteoclasts, endothelial cells and pericytes [6]. BMPs are the most investigated growth factors involved in bone regeneration. BMPs diversely affect osteoblast lineage cells and stimulate the proliferation and differentiation of mesenchymal progenitors, preosteoblasts, and osteoblasts [39, 40]. Particularly, BMP2 recruits bone-lining cells at defected regions and enhances their differentiation by modulating angiogenesis [41, 42]. Since its FDA approval in 2002, recombinant human BMP2 (rhBMP2) has been used for treating patients with various bone defects [39]. Interestingly, CME did not contain BMP2, as determined by ELISA (Fig 4). However, osteogenic effect of CME showed more promising results than BMP2 in the present study (Fig 9). Treatment with 200–400 μg/mL CME might provide an appropriate environment for the differentiation of MG-63 cells, which reached the late stage of osteogenesis on day 7 and 9 after treatment (Fig 2). These results indicate that CME constituents promote the osteogenesis of MG-63 cells. A further study is needed to identify the exact component.

With BMP2, growth factors such as bFGF, TGFβ-1 and EGF play important roles in bone regeneration by promoting osteoblast proliferation and differentiation. In our study, treatment with high BMP2 doses induced ECM mineralization after 18 days; however, the combination of BMP2 + FGF2 + TGFβ-1 did not promote the late-stage differentiation of MG-63 cells (Fig 9 and S4 Fig). However, Alizarin red S staining demonstrated the significant efficacy of CME in promoting the mineralization of MG-63 cells compared with that of growth factors alone and/or their combinations (Fig 9). These results indicated that CME possess potential for bone formation even in the absence of BMP2. In addition, interplay among different growth factors may influence stimulatory or inhibitory effects of osteoblasts which may differ among the combination and ratio of growth factors.

In previous study, we optimized the in vitro dose of BMP2 as 500 ng/mL for enhancing osteogenesis on MG-63 cells at 20 days after the treatment in OIM. Therefore, here we used 500 ng/mL BMP2 as the highest concentration in single growth factor treatment for increasing mineralized nodule formation on MG-63 cells (Fig 9). Previous studies reported much lower concentration of growth factors for in vitro osteogenic differentiation on human and mouse MSCs [7, 43]. The optimum dosage of growth factors for in vitro osteogenesis may vary by the cell type used in the experimental system. For example, in our study lower concentration of bFGF have shown an increased ALP activity hMSCs at day 7, however, no effect on MG-63 cells (Fig 9). The reason could be the different ability of cells as an osteoblast on in vitro osteogenic differentiation. In addition, the promoting effect of growth factors on osteogenesis may differ by the period of in vitro ostoegenesis. Many studies commonly used low growth factor doses and long culture period for in vitro differentiation of osteoblasts. Here, in order to detect the stimulatory effect of CME on osteogenesis during short differentiation period, we treated high concentration of growth factors and compared the efficacy of osteogenesis. Our results showed that CME significantly promoted mineralization and calcium accumulation only at 7 and 9 days after osteogenic induction while high doses of growth factors could not enhance the osteogenesis of MG 63 cells. Therefore, the potential ability of CME for osteogenesis was much better than several bone-specific growth factors, even if high concentration of growth factors were used under the same experimental condition and period.

Growth factors are known to be involved in proliferation and differentiation of osteo-progenitor cells. Previous data showed that osteogenesis of rat MSCs was promoted by adding bFGF in the earlier stages followed by the treatment of BMP2 at the later stage [7]. They determined the temporal and synergistic effect of bFGF and BMP2 on osteogenesis and explained the reason; the treatment of bFGF at the early stage increased the total number of cells, which may also increase the BMP2 receptors on the cell surfaces to enhance the binding potential with added BMP2 at the late stage. In this study, our main focus was to study the differentiation effects of growth factors on osteogenesis of MG-63 cells, rather than the proliferation effects. We constantly applied growth factors to study the osteogenic effects on MG63 cells and compared with CME. The temporal and multiple combination of growth factors for osteogenesis will consider in our future experiments. Furthermore, the optimal time for growth factor addition and dosages during osteogenesis further needs to study to understand the natural spatiotemporal process of bone regeneration. Altogether, our results showed that CME is a complete natural solution that is easier to apply to enhance osteogenesis than selectively addition of bone-specific growth factors during differentiation of osteoblasts.

Recent studies have suggested that continuous treatment with rhBMP2 leads to adverse such as carcinogenesis and excessive bone regeneration [39, 44, 45]. Therefore, CME might be an excellent alternative to BMP2 for bone regeneration because it is easy to obtain, is more safe and biocompatible for *in vivo* application, and promote the osteogenic differentiation of osteoblasts.

The bFGF has biological activities including proliferation, migration, angiogenesis, and bone differentiation. However, the osteogenic effects of bFGF on osteoblasts are controversial. Dombrowski et al. have shown that blocking of FGF signaling using the chemical inhibitor SU5402 increases mineralization of rat bone marrow stem cells (rMSCs) in OIM for 21 days [46]. However, we cultured MG-63 cells in OIM with SU5402 for only 9 days. No detectable change of mineralization was observed during 9 days on MG-63 cells cultured in OIM containing SU5402 (S3 Fig). Collectively, it implies that FGF signaling does not affect osteogenic differentiation at the early stage of osteogenesis whereas this specific signaling has negative effect on the late stage of osteogenic differentiation, suggesting the continuous activation of FGF signaling may be unnecessary for osteogenic differentiation in *in vitro* system. In addition, a new fining recently supported by Kim et al., identified DJ-1 protein which is a novel osteogenic factor to promote the osteogenesis of MSCs through the activation of FGF signaling [47]. We have shown the inhibitory effect of SU5402 on the osteogenic differentiation of CME-treated MG-63 cells (Fig 5), indicating that CME may contain an osteogenic stimulator to enhance mineralization of osteoblast-like cells via FGF signaling like DJ-1.

In the present study, we evaluated the effects of CME on the differentiation of osteoblast-like MG-63 cells and hMSCs. The effect of CME on other bone lineage cells and different types of osteoblasts *in vitro* and *in vivo* needs to be evaluated along with the mechanism underlying the stimulation of osteogenesis by CME.

## Conclusion

**Fig 10:**
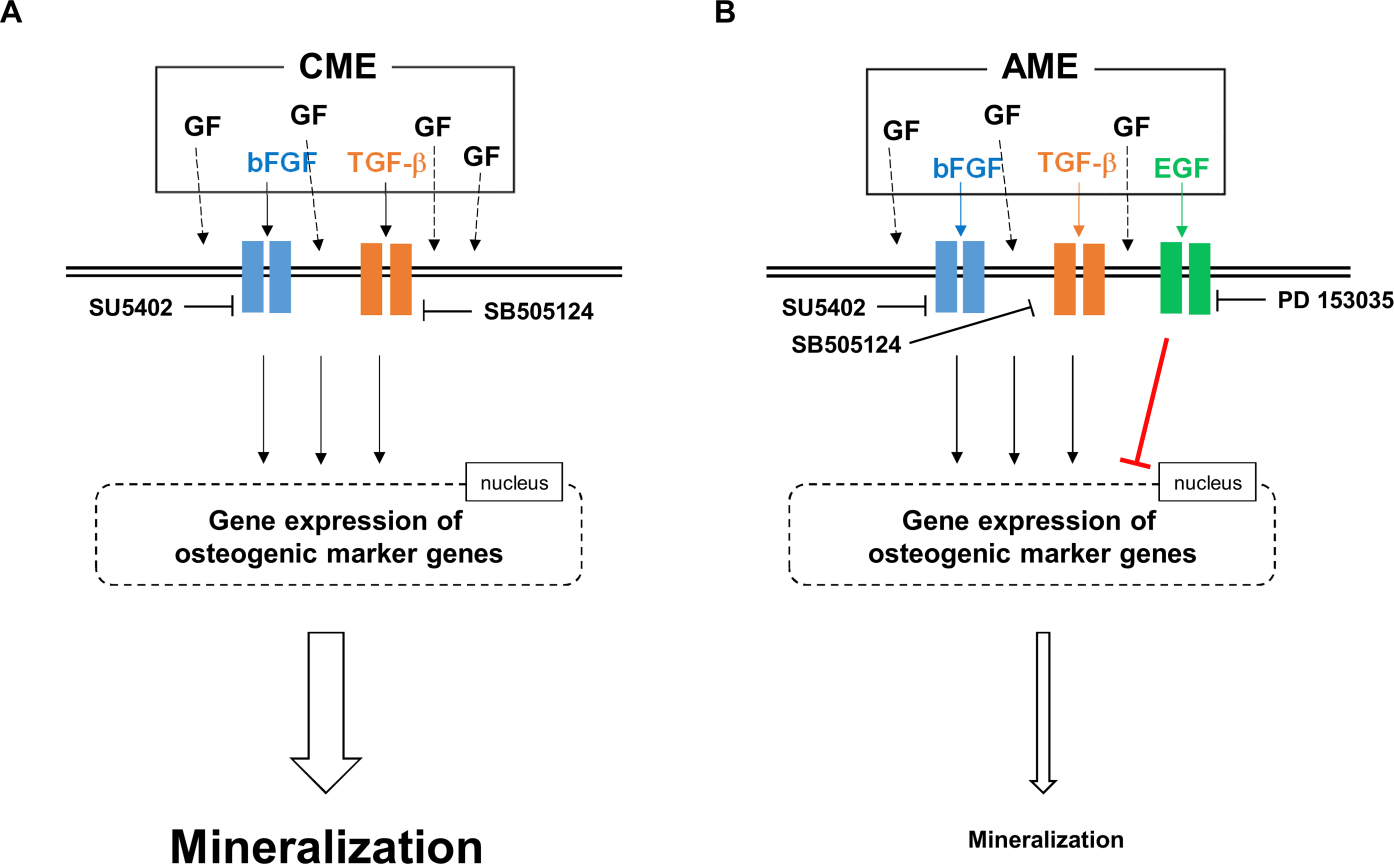
Proposed mechanisms underlying the effect of growth factors present in AME and CME on the osteogenic differentiation of MG-63 cells. (A) Of the several growth factors present in CME, bFGF and TGFβ-1 positively regulated the expression of osteogenic marker genes, which subsequently promoted ECM mineralization during the osteogenic differentiation of MG-63 cells. (B) AME contained high concentrations of EGF along with bFGF and TGFβ-1. bFGF and TGFβ-1 upregulated the expression of osteogenic marker genes, which positively affect to the formation of mineralization, like CME. In contrast, activation of EGF signaling in AME-treated MG-63 cells decreased the expression of osteogenic marker genes, thus preventing these cells from entering the late stage of osteogenic differentiation, which is characterized by ECM mineralization.

Both AME and CME enhanced the osteogenesis of MG-63 cells; however, CME was more effective in promoting osteogenesis than AME. We found that bFGF and TGFβ-1 present in both the extracts positively regulated the osteogenic differentiation of MG-63 cells while EGF present in AME negatively regulated the osteogenic differentiation of MG-63 cells (Fig 10). Based on their growth factor composition, AME and CME exerted differential effects on the expression of osteogenic marker genes, which in turn affected the mineralization of MG-63 cells during osteogenesis (Fig 10). Moreover, our results clearly indicated the excellent capacity of CME in promoting bone regeneration compared with that bFGF, TGFβ-1, and BMP2 alone or in combination. Therefore, we suggest two possible translational approaches to various bone disease such as osteoporosis and osteopetrosis. The first strategy is a combination treatment of CME with stem cells which may use in bone defect to promote bone growth and healing. On the other hand, AME may provide a new therapeutic strategy to modulate the bone density or calcification during bone regeneration by modifying osteogenic efficacy of AME using EGFR inhibitors.

The exact regulatory mechanisms underlying the mineralization of osteoblasts induced by growth factors and proteins in the human amniotic membrane are unclear. However, our findings clearly indicate the potential of CME in promoting the osteogenic differentiation of osteoblast-like cells and human bone marrow derived MSCs, suggesting that it can be used as a novel therapeutic biomaterial for bone regeneration.

## Supporting information

S1 Fig Image of human amniotic membranes and extracts. (A) Representative images showed amnion, chorion, and conjoined amnion/chorion membrane. (B) AME and CME were prepared as described in Material and Method section shown here.
(TIF)

S2 Fig Osteogenesis of MG-63 cells. (A) Expression profiles of ALP activity were determined during MG-63 osteogenesis. GM: growth medium and OIM: Osteogenesis Induction Medium. (B) Calcium contents of MG-63 cells during osteogenesis were analyzed by performing the calcium assay at day 7 after the induction. (C) The mRNA levels of osteogenic markers, *ALP*, *IBSP*, *RUNX2*, *OSTERIX* and *OCN* were examined by quantitative RT-PCR about 4 days after the induction of osteogenesis. *p< 0.01 and **p< 0.001, Student’s t-test. Data are presented as the mean ± SD of multiple repeated experiments.
(TIF)

S3 Fig Suramin sodium, SU5402, and SB505124 did not affect the osteogenesis of MG-63 cells. The experiment performed without CME treatment at the same condition described (A) in Fig 2A, (B) in Fig 4A, (C) in Fig 4B, and (D) in Fig 5A, respectively. Data are presented as the mean ± SD of multiple repeated experiments.
(TIF)

S4 Fig Recombinant human growth factors did not induce the mineralization of MG-63 cells. The experimental condition described in Fig 7. Microscope images showed MG-63 cells cultured in OIM with bFGF (500 ng/mL), TGFβ-1 (500 ng/mL), CME (100 μg/mL), bFGF + TGFβ-1 (500 ng/mL of each growth factor), and bFGF + TGFββ-1 + BMP2 (500 ng/mL of each growth factor). Scale bars represent 500 μm.
(TIF)

S1 Table List of primers and product sizes for quantitative real-time RT-PCR. (DOCX)

## References

[1] Karsenty G, Wagner EF: Reaching a Genetic and Molecular Understanding of Skeletal Development. Developmental Cell 2002, 2(4):389–406. 11970890 10.1016/s1534-5807(02)00157-0

[2] Blair HC, Sun LI, Kohanski RA: Balanced Regulation of Proliferation, Growth, Differentiation, and Degradation in Skeletal Cells. Annals of the New York Academy of Sciences 2007, 1116(1):165–173. 17646258 10.1196/annals.1402.029

[3] Blair HC, Zaidi M, Huang CLH, Sun L: The Developmental Basis of Skeletal Cell Differentiation and the Molecular Basis of Major Skeletal Defects. Biological Reviews 2008, 83(4):401–415. doi: 10.1111/j.1469-185X.2008.00048.x 18710437 10.1111/j.1469-185X.2008.00048.x

[4] Pittenger MF, Mackay AM, Beck SC, Jaiswal RK, Douglas R, Mosca JD et al: Multilineage Potential of Adult Human Mesenchymal Stem Cells. Science 1999, 284(5411):143–147. 10102814 10.1126/science.284.5411.143

[5] Ducy P, Schinke T, Karsenty G: The Osteoblast: A Sophisticated Fibroblast under Central Surveillance. Science 2000, 289(5484):1501–1504. 10968779 10.1126/science.289.5484.1501

[6] Long F: Building strong bones: molecular regulation of the osteoblast lineage. Nat Rev Mol Cell Biol 2012, 13(1):27–38. 10.1038/nrm3254 22189423

[7] Huang Z, Ren PG, Ma T, Smith RL, Goodman SB: Modulating osteogenesis of mesenchymal stem cells by modifying growth factor availability. Cytokine 2010, 51(3):305–310. doi: 10.1016/j.cyto.2010.06.002 20580248 10.1016/j.cyto.2010.06.002

[8] Mitchell AC, Briquez PS, Hubbell JA, Cochran JR: Engineering growth factors for regenerative medicine applications. Acta Biomaterialia. 10.1016/j.actbio.2015.11.007 PMC6067679 26555377

[9] Kang M, Choi S, Cho Lee A-R: Effect of freeze dried bovine amniotic membrane extract on full thickness wound healing. Arch Pharm Res 2013, 36(4):472–478. doi: 10.1007/s12272-013-0079-5 23512774 10.1007/s12272-013-0079-5

[10] Hao Y, Ma DH-K, Hwang DG, Kim W-S, Zhang F: Identification of Antiangiogenic and Antiinflammatory Proteins in Human Amniotic Membrane. Cornea 2000, 19(3):348–352. 10832697 10.1097/00003226-200005000-00018

[11] Sangwan VS, Basu S: Antimicrobial properties of amniotic membrane. British Journal of Ophthalmology 2011, 95(1):1–2. doi: 10.1136/bjo.2010.184259 21163818 10.1136/bjo.2010.184259

[12] Go YY, Kim SE, Cho GJ, Chae SW, Song JJ: Promotion of osteogenic differentiation by amnion/chorion membrane extracts. Journal of applied biomaterials & functional materials 2016, 14(2):e171–180. 27056480 10.5301/jabfm.5000264

[13] Luu HH, Song W-X, Luo X, Manning D, Luo J, Deng Z-L et al: Distinct roles of bone morphogenetic proteins in osteogenic differentiation of mesenchymal stem cells. Journal of Orthopaedic Research 2007, 25(5):665–677. doi: 10.1002/jor.20359 17290432 10.1002/jor.20359

[14] Miraoui H, Oudina K, Petite H, Tanimoto Y, Moriyama K, Marie PJ: Fibroblast Growth Factor Receptor 2 Promotes Osteogenic Differentiation in Mesenchymal Cells via ERK1/2 and Protein Kinase C Signaling. Journal of Biological Chemistry 2009, 284(8):4897–4904. doi: 10.1074/jbc.M805432200 19117954 10.1074/jbc.M805432200

[15] Ozkan K, Eralp L, Kocaoglu M, Ahishali B, Bilgic B, Mutlu Z et al: The effect of transforming growth factor β1 (TGF-β1) on the regenerate bone in distraction osteogenesis. Growth Factors 2007, 25(2):101–107. doi: 10.1080/08977190701352594 17891595 10.1080/08977190701352594

[16] Tamama K, Kawasaki H, Wells A: Epidermal Growth Factor (EGF) Treatment on Multipotential Stromal Cells (MSCs). Possible Enhancement of Therapeutic Potential of MSC. Journal of Biomedicine and Biotechnology 2010, 2010:10. 10.1155/2010/795385 PMC2825653 20182548

[17] Li RD, Deng ZL, Hu N, Liang X, Liu B, Luo J et al: Biphasic effects of TGFbeta1 on BMP9-induced osteogenic differentiation of mesenchymal stem cells. BMB reports 2012, 45(9):509–514. 23010171 10.5483/bmbrep.2012.45.9.053

[18] Li XL, Liu YB, Ma EG, Shen WX, Li H, Zhang YN: Synergistic effect of BMP9 and TGF-beta in the proliferation and differentiation of osteoblasts. Genetics and molecular research: GMR 2015, 14(3):7605–7615. doi: 10.4238/2015.July.13.4 26214439 10.4238/2015.July.13.4

[19] Gurkan UA, Gargac J, Akkus O: The sequential production profiles of growth factors and their relations to bone volume in ossifying bone marrow explants. Tissue engineering Part A 2010, 16(7):2295–2306. doi: 10.1089/ten.TEA.2009.0565 20184436 10.1089/ten.TEA.2009.0565

[20] Compton J, Fragomen A, Rozbruch SR: Skeletal Repair in Distraction Osteogenesis: Mechanisms and Enhancements. JBJS Reviews 2015, 3(8). 10.2106/JBJS.RVW.N.00107 27490473

[21] Yun Y-P, Lee S-Y, Kim H-J, Song J-J, Kim S: Improvement of osteoblast functions by sustained release of bone morphogenetic protein-2 (BMP-2) from heparin-coated chitosan scaffold. Tissue Eng Regen Med 2013, 10(4):183–191.

[22] Salem O, Wang H, Alaseem A, Ciobanu O, Hadjab I, Gawri R et al: Naproxen affects osteogenesis of human mesenchymal stem cells via regulation of Indian hedgehog signaling molecules. Arthritis Research & Therapy 2014, 16(4):R152. 25034046 10.1186/ar4614 PMC4223691

[23] Song S, Wientjes MG, Gan Y, Au JL: Fibroblast growth factors: an epigenetic mechanism of broad spectrum resistance to anticancer drugs. Proceedings of the National Academy of Sciences of the United States of America 2000, 97(15):8658–8663. doi: 10.1073/pnas.140210697 10890892 10.1073/pnas.140210697 PMC27004

[24] Coffey RJ Jr., Leof EB, Shipley GD, Moses HL: Suramin inhibition of growth factor receptor binding and mitogenicity in AKR-2B cells. J Cell Physiol 1987, 132(1):143–148. doi: 10.1002/jcp.1041320120 3496343 10.1002/jcp.1041320120

[25] Kloen P, Jennings CL, Gebhardt MC, Springfield DS, Mankin HJ: Suramin inhibits growth and transforming growth factor-β1 (TGF-β1) binding in osteosarcoma cell lines. European Journal of Cancer 1994, 30(5):678–682. 10.1016/0959-8049(94)90544-4 8080687

[26] Lu J, Dai J, Wang X, Zhang M, Zhang P, Sun HAO et al: Effect of fibroblast growth factor 9 on the osteogenic differentiation of bone marrow stromal stem cells and dental pulp stem cells. Molecular Medicine Reports 2015, 11(3):1661–1668. doi: 10.3892/mmr.2014.2998 25435023 10.3892/mmr.2014.2998 PMC4270321

[27] Suzuki E, Ochiai-Shino H, Aoki H, Onodera S, Saito A, Saito A et al: Akt Activation is Required for TGF-β1-Induced Osteoblast Differentiation of MC3T3-E1 Pre-Osteoblasts. PLoS ONE 2014, 9(12):e112566 doi: 10.1371/journal.pone.0112566 25470129 10.1371/journal.pone.0112566 PMC4254279

[28] Khan SN, Lane JM: The use of recombinant human bone morphogenetic protein-2 (rhBMP-2) in orthopaedic applications. Expert opinion on biological therapy 2004, 4(5):741–748. doi: 10.1517/14712598.4.5.741 15155165 10.1517/14712598.4.5.741

[29] Lee K, Silva EA, Mooney DJ: Growth factor delivery-based tissue engineering: general approaches and a review of recent developments. Journal of the Royal Society, Interface 2011, 8(55):153–170. 10.1098/rsif.2010.0223 PMC3033020 20719768

[30] Recombinant human bone morphogenetic protein-2 stimulates osteoblastic maturation and inhibits myogenic differentiation in vitro. The Journal of Cell Biology 1991, 113(3):681–687. 1849907 10.1083/jcb.113.3.681 PMC2288971

[31] Wu L-A, Wang F, Donly KJ, Baker A, Wan C, Luo D et al: Establishment of Immortalized BMP2/4 Double Knock-Out Osteoblastic Cells is Essential for Study of Osteoblast Growth, Differentiation and Osteogenesis. Journal of Cellular Physiology 2015(9999):1–10. 26595646 10.1002/jcp.25266 PMC4784166

[32] Takuwa Y, Ohse C, Wang EA, Wozney JM, Yamashita K: Bone morphogenetic protein-2 stimulates alkaline phosphatase activity and collagen synthesis in cultured osteoblastic cells, MC3T3-E1. Biochemical and Biophysical Research Communications 1991, 174(1):96–101. 1989624 10.1016/0006-291x(91)90490-x

[33] Sakou T: Bone Morphogenetic Proteins: From Basic Studies to Clinical Approaches. Bone 1998, 22(6):591–603. 9626397 10.1016/s8756-3282(98)00053-2

[34] Sorsby A, Haythorne J, Reed H: FURTHER EXPERIENCE WITH AMNIOTIC MEMBRANE GRAFTS IN CAUSTIC BURNS OF THE EYE. The British Journal of Ophthalmology 1947, 31(7):409–418. 18170361 10.1136/bjo.31.7.409 PMC513860

[35] Dua HS, Rahman I, Miri A, Said DG: Variations in amniotic membrane: relevance for clinical applications. British Journal of Ophthalmology 2010, 94(8):963–964. doi: 10.1136/bjo.2009.157941 20679087 10.1136/bjo.2009.157941

[36] Gholipourmalekabadi M, Bandehpour M, Mozafari M, Hashemi A, Ghanbarian H, Sameni M et al: Decellularized human amniotic membrane: more is needed for an efficient dressing for protection of burns against antibiotic-resistant bacteria isolated from burn patients. Burns 2015, 41(7):1488–1497. doi: 10.1016/j.burns.2015.04.015 26048133 10.1016/j.burns.2015.04.015

[37] Rennie K, Gruslin A, Hengstschläger M, Pei D, Cai J, Nikaido T et al: Applications of Amniotic Membrane and Fluid in Stem Cell Biology and Regenerative Medicine. Stem Cells International 2012, 2012:721538 doi: 10.1155/2012/721538 23093978 10.1155/2012/721538 PMC3474290

[38] Koizumi NJ, Inatomi TJ, Sotozono CJ, Fullwood NJ, Quantock AJ, Kinoshita S: Growth factor mRNA and protein in preserved human amniotic membrane. Current eye research 2000, 20(3):173–177. 10694891

[39] Carreira AC, Lojudice FH, Halcsik E, Navarro RD, Sogayar MC, Granjeiro JM: Bone Morphogenetic Proteins: Facts, Challenges, and Future Perspectives. Journal of Dental Research 2014, 93(4):335–345. doi: 10.1177/0022034513518561 24389809 10.1177/0022034513518561

[40] Garrison KR, Shemilt I, Donell S, Ryder JJ, Mugford M, Harvey I et al: Bone morphogenetic protein (BMP) for fracture healing in adults. The Cochrane database of systematic reviews 2010(6):Cd006950 doi: 10.1002/14651858.CD006950.pub2 20556771 10.1002/14651858.CD006950.pub2 PMC6669254

[41] Peng H, Usas A, Olshanski A, Ho AM, Gearhart B, Cooper GM et al: VEGF Improves, Whereas sFlt1 Inhibits, BMP2-Induced Bone Formation and Bone Healing Through Modulation of Angiogenesis. Journal of Bone and Mineral Research 2005, 20(11):2017–2027. 10.1359/JBMR.050708 16234975

[42] Choi H, Jeong B-C, Hur S-W, Kim J-W, Lee K-B, Koh J-T: The Angiopoietin-1 Variant COMP-Ang1 Enhances BMP2-Induced Bone Regeneration with Recruiting Pericytes in Critical Sized Calvarial Defects. PLoS ONE 2015, 10(10):e0140502 doi: 10.1371/journal.pone.0140502 26465321 10.1371/journal.pone.0140502 PMC4605622

[43] Lee JH, Jang SJ, Baek HR, Lee KM, Chang BS, Lee CK: Synergistic induction of early stage of bone formation by combination of recombinant human bone morphogenetic protein-2 and epidermal growth factor. Journal of tissue engineering and regenerative medicine 2015, 9(4):447–459. doi: 10.1002/term.1900 24764222 10.1002/term.1900 PMC4497359

[44] Carragee EJ, Hurwitz EL, Weiner BK: A critical review of recombinant human bone morphogenetic protein-2 trials in spinal surgery: emerging safety concerns and lessons learned. The Spine Journal 2011, 11(6):471–491. doi: 10.1016/j.spinee.2011.04.023 21729796 10.1016/j.spinee.2011.04.023

[45] Lad SP, Bagley JH, Karikari IO, Babu R, Ugiliweneza B, Kong M et al: Cancer After Spinal Fusion: The Role of Bone Morphogenetic Protein. Neurosurgery 2013, 73(3):440–449. doi: 10.1227/NEU.0000000000000018 23756740 10.1227/NEU.0000000000000018

[46] Dombrowski C, Song SJ, Chuan P, Lim X, Susanto E, Sawyer AA et al: Heparan sulfate mediates the proliferation and differentiation of rat mesenchymal stem cells. Stem cells and development 2009, 18(4):661–670. doi: 10.1089/scd.2008.0157 18690792 10.1089/scd.2008.0157

[47] Kim JM, Shin HI, Cha SS, Lee CS, Hong BS, Lim S et al: DJ-1 promotes angiogenesis and osteogenesis by activating FGF receptor-1 signaling. Nature communications 2012, 3:1296 doi: 10.1038/ncomms2313 23250426 10.1038/ncomms2313

