# Supplementary figures and images for: Differential effects of amnion and chorion membrane extracts on osteoblast-like cells due to the different growth factor composition of the extracts
Source: PLoS One. 2017 Aug 10;12(8):e0182716. doi: 10.1371/journal.pone.0182716 (PMC5552222; doi:10.1371/journal.pone.0182716)

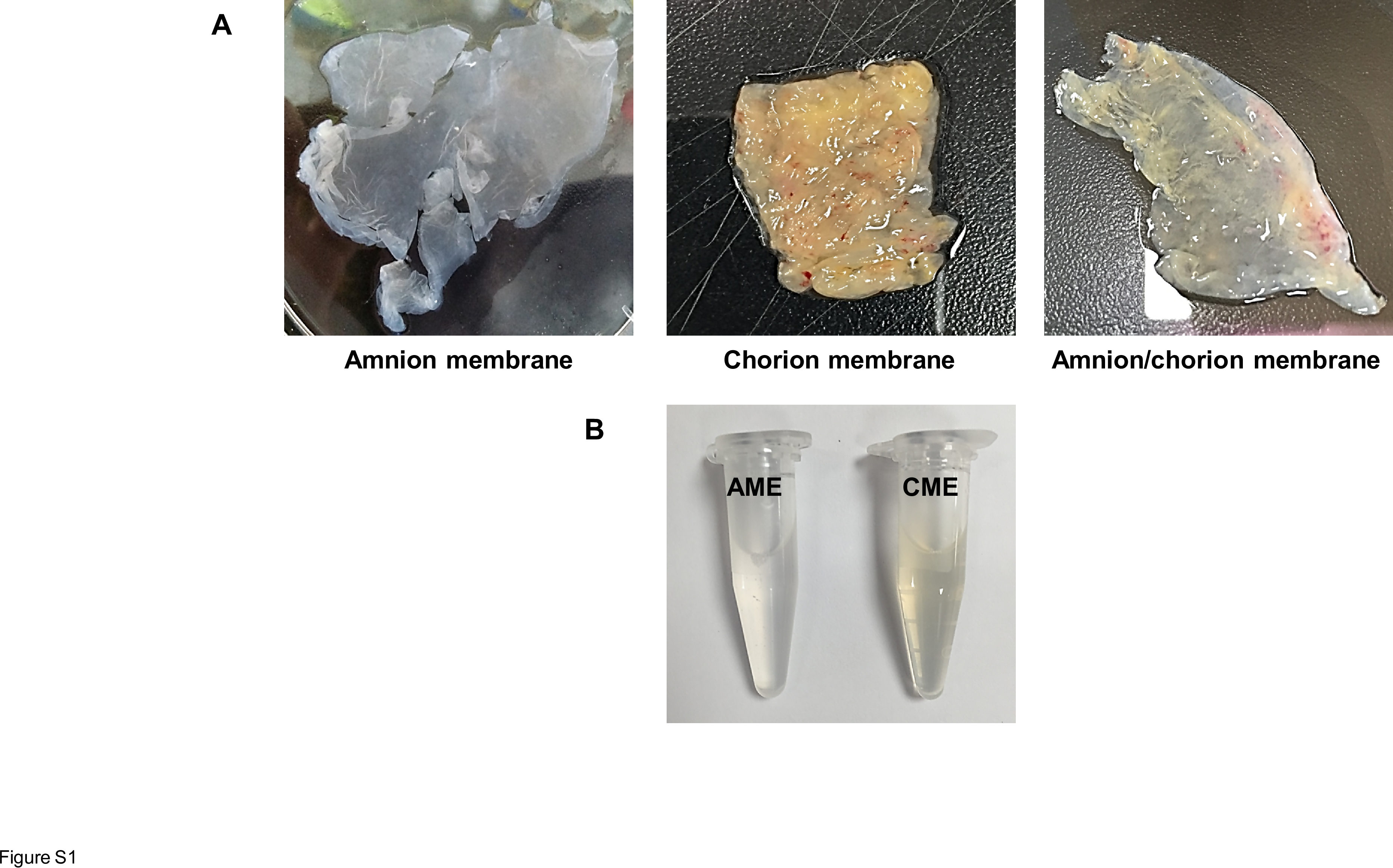

Supplement: S1 Fig — (A) Representative images showed amnion, chorion, and conjoined amnion/chorion membrane. (B) AME and CME were prepared as described in Material and Method section shown here. (TIF) [file pone.0182716.s001.tif]

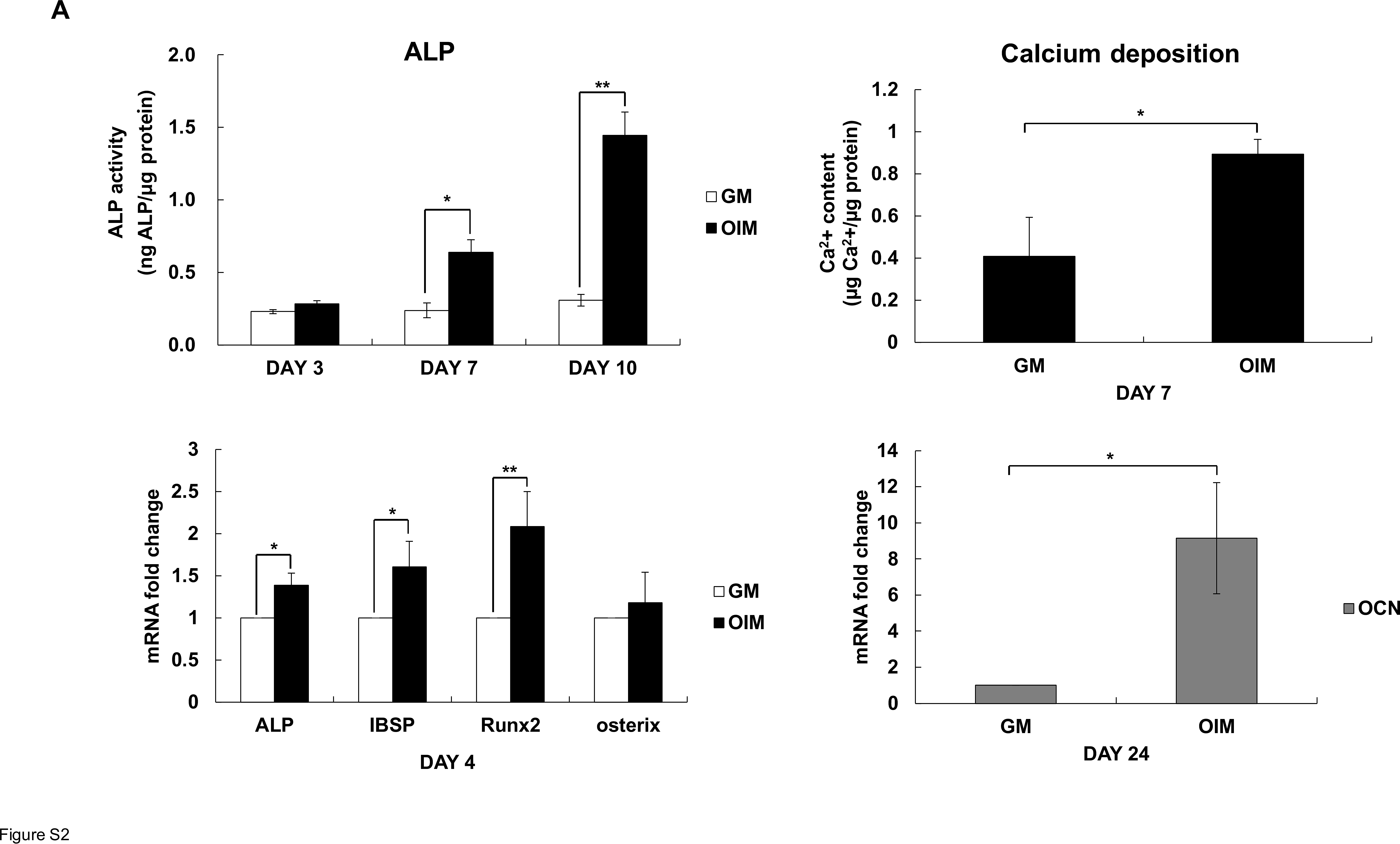

Supplement: S2 Fig — (A) Expression profiles of ALP activity were determined during MG-63 osteogenesis. GM: growth medium and OIM: Osteogenesis Induction Medium. (B) Calcium contents of MG-63 cells during osteogenesis were analyzed by performing the calcium assay at day 7 after the induction. (C) The mRNA levels of osteogenic markers, ALP, IBSP, RUNX2, OSTERIX and OCN were examined by quantitative RT-PCR about 4 days after the induction of osteogenesis. *p< 0.01 and **p< 0.001, Student’s t-test. Data are presented as the mean ± SD of multiple repeated experiments. (TIF) [file pone.0182716.s002.tif]

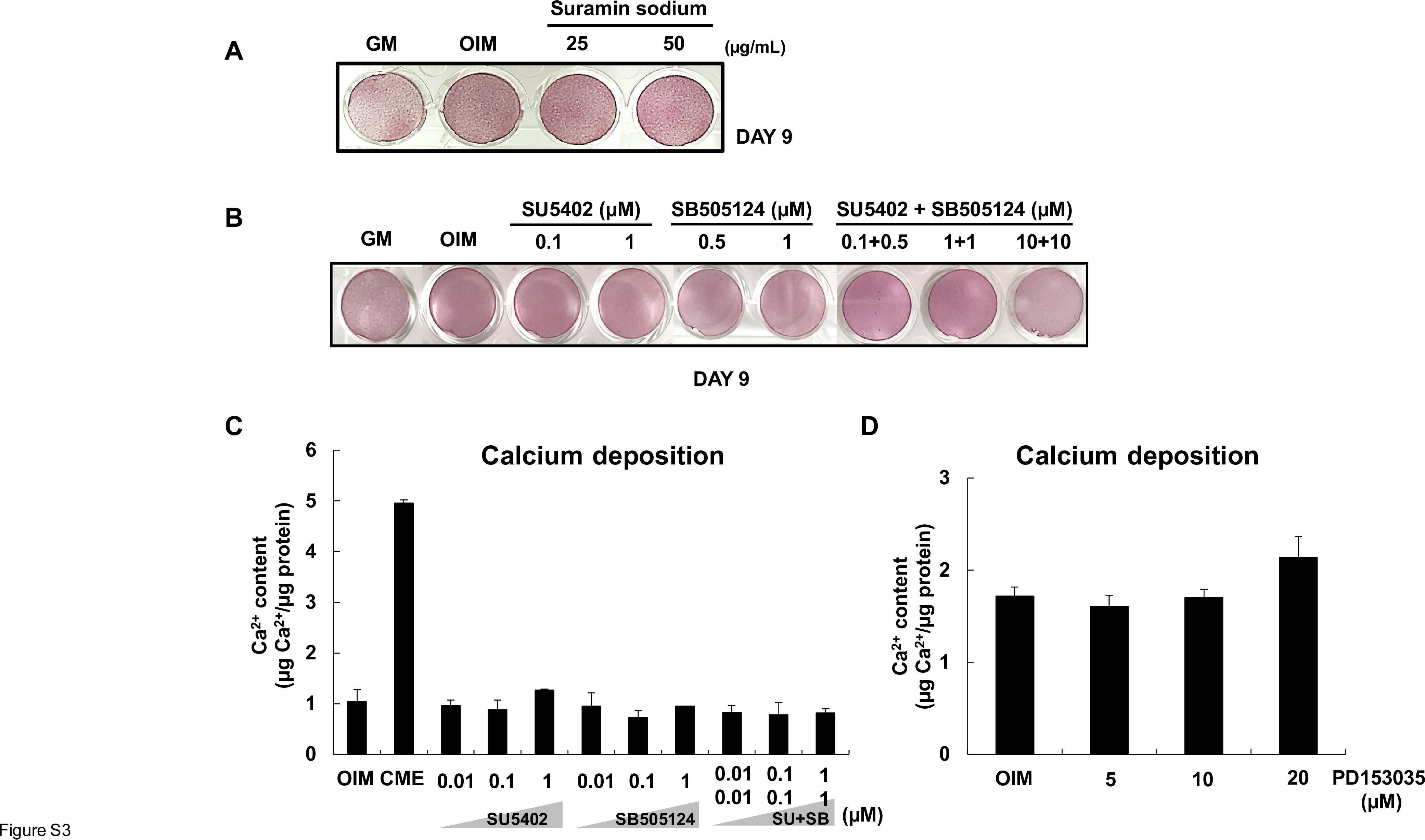

Supplement: S3 Fig — The experiment performed without CME treatment at the same condition described (A) in Fig 2A, (B) in Fig 4A, (C) in Fig 4B, and (D) in Fig 5A, respectively. Data are presented as the mean ± SD of multiple repeated experiments. (TIF) [file pone.0182716.s003.tif]

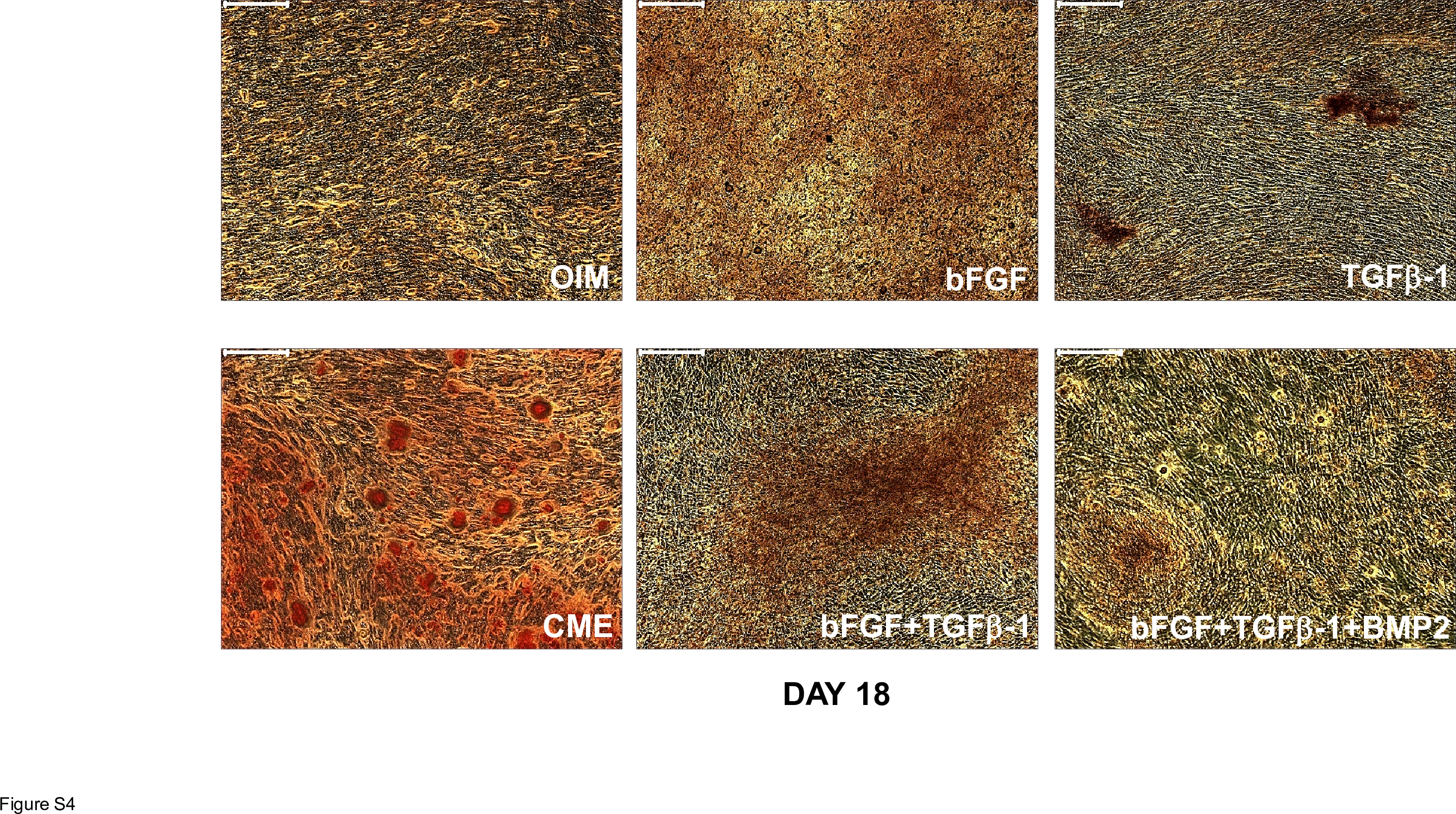

Supplement: S4 Fig — The experimental condition described in Fig 7. Microscope images showed MG-63 cells cultured in OIM with bFGF (500 ng/mL), TGFβ-1 (500 ng/mL), CME (100 μg/mL), bFGF + TGFβ-1 (500 ng/mL of each growth factor), and bFGF + TGFββ-1 + BMP2 (500 ng/mL of each growth factor). Scale bars represent 500 μm. (TIF) [file pone.0182716.s004.tif]
