# Supplementary material for: Differential effects of amnion and chorion membrane extracts on osteoblast-like cells due to the different growth factor composition of the extracts
Source: PLoS One. 2017 Aug 10;12(8):e0182716. doi: 10.1371/journal.pone.0182716 (PMC5552222; doi:10.1371/journal.pone.0182716)
Supplement: S1 Table — (DOCX) [file pone.0182716.s005.docx]

| **Gene name** | **Primer sequence** | **Product size (bp)** |
| --- | --- | --- |
| ***ALP*** | F-TAACATCAGGGACATTGACG | 165 |
|  | R-TGCTTGTATCTCGGTTTGAA |  |
| ***RUNX2*** | F-GACACCACCAGGCCAATC | 125 |
|  | R-AGAACAAGGGGGCCGTTA |  |
| ***OSTERIX*** | F-GCCAGAAGCTGTGAAACCTC | 121 |
|  | R-TGATGGGGTCATGGTGTCTA |  |
| ***OCN*** | F-TGACGAGTTGGCTGACCA | 56 |
|  | R-GCCGTAGAAGCGCCGATAGGC |  |
| ***IBSP*** | F-GAACAAGGCATAAACGGCACC | 120 |
|  | R-TTCTGCATTGGCTCCAGTGAC |  |
| ***GAPDH*** | F-TCGCCCCACTTGATTTTGG | 105 |
|  | R-GCAAATTCCATGGCACCGT |  |

S5 Table. List of primers and product sizes for quantitative real-time RT-PCR.
